# Human–Machine Collaboration for Accelerated Discovery of Promising Oxygen Evolution Electrocatalysts with On-Demand Elements

**DOI:** 10.1021/acscentsci.3c01009

**Published:** 2023-11-30

**Authors:** Ken Sakaushi, Watcharaporn Hoisang, Ryo Tamura

**Affiliations:** † Research Center for Energy and Environmental Materials, National Institute for Materials Science, 1-1 Namiki, Tsukuba, Ibaraki 305-0044, Japan; ‡ Center for Basic Research on Materials, National Institute for Materials Science, 1-1 Namiki, Tsukuba, Ibaraki 305-0044, Japan; § Graduate School of Frontier Sciences, The University of Tokyo, Kashiwa 277-8561, Japan

## Abstract

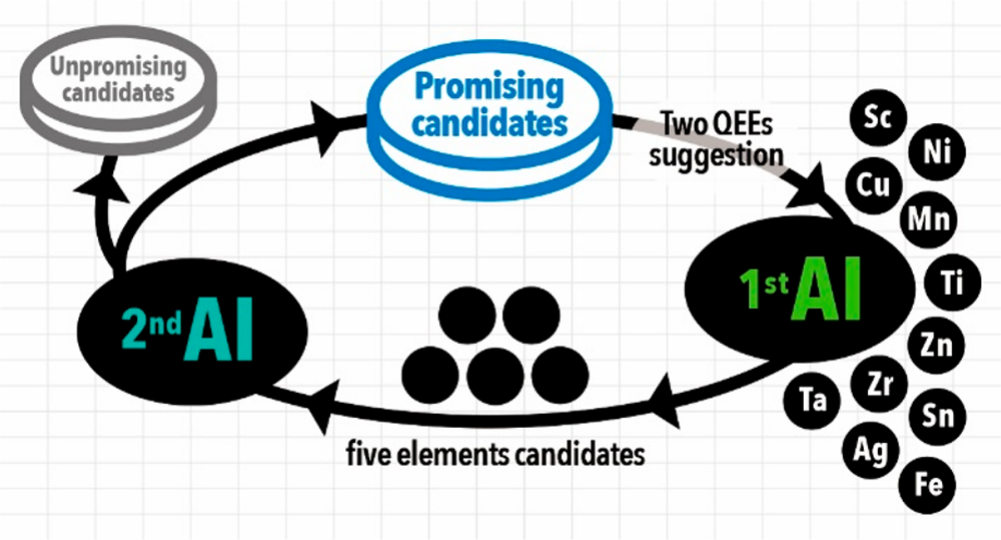

A drastically efficient method for identifying electrocatalysts with desirable functionality is a pressing necessity for making a breakthrough in advanced water-electrolyzers toward large-scale green hydrogen production and addressing the significant challenge of carbon neutrality. Despite extensive investigations over the last several centuries, it remains a time-consuming task to identify even one promising affordable electrocatalyst without platinum-group-metal (PGM) for one electrochemical reaction due to its great complexities, particularly for the key anode reaction in the water-electrolyzer of the oxygen evolution reaction (OER). In this study, we demonstrate that a human–machine collaboration based on stepwise-evolving artificial intelligence (se-AI) can significantly shorten the development period of PGM-free multimetal OER electrocatalysts with performance beyond a PGM of RuO_2_. We were able to reach optimized materials only after 2% experimental trials of the entire candidate pool. The best PGM-free electrocatalyst discovered exhibited excellent activity comparable to RuO_2_ and, surprisingly, also demonstrated superior stability with a high current density of up to 1000 mA/cm^2^ at even pH 9.2, which condition is a thermodynamically challenging for typical PGM-free materials. This work illustrates that human’s material discovery can be significantly accelerated through collaboration with AI.

## Abstract 2

A specially designed human−machine collaboration shortened development time, achieving promising PGM-free oxygen evolution electrocatalysts, accelerating material discovery with on-demand elements.

## Introduction

Several decades are typically required to identify an optimized material for a specific device.^1−4^ This period, encompassing material discovery and trial-and-error-based optimization, is known to be a major lead time in the implementation of key technologies in our society. Thus, an approach that can significantly accelerate the discovery of materials with desirable functions is imperative. In recent years, a huge number of artificial intelligence (AI) based attempts have been made to speed up the process of finding materials with desired properties, to shorten this time-consuming step, and establish advanced devices more efficiently.^5−9^ This AI-based method is of particular interest for the advancement of water-splitting devices, where the oxygen evolution reaction (OER) electrocatalyst is a key material, to achieve carbon-neutrality. However, the electrochemical reaction is complex, and thus a groundbreaking model study is necessary to improve the methodology for using AI in electrocatalyst research.^10,11^

**Figure 1:**
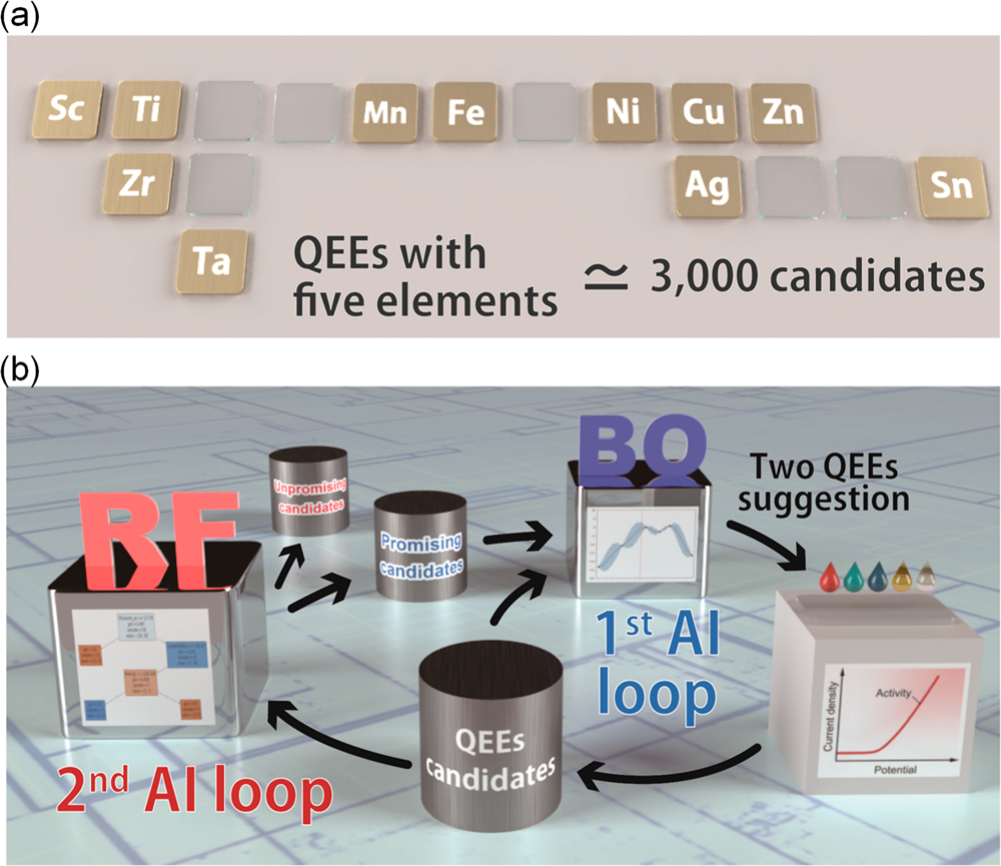
The framework of the human–machine collaboration for accelerated OER electrocatalyst discovery. (a) Selected PGM-free 11 elements for preparing QEEs. (b) Schematics of AI–human collaboration using our stepwise-evolving artificial intelligence (se-AI). In the first loop of the AI process (1st AI loop), quinary-element electrocatalyst (QEE) candidates are selected using Bayesian optimization (BO). Based on the selection from the first loop, humans synthesize suggested materials and evaluate their electrochemical properties. Once a sufficient amount of data has been collected, the 1st AI loop is upgraded to the 2nd AI loop, which is integrated with the Random Forest (RF) classifier and BO. In the second loop, the collected data is classified by the RF and separated into promising and unpromising groups. The data from the promising group is then applied to BO to obtain suggestions for synthesizing better OER electrocatalysts. This stepwise evolution of AI allows for accelerated discovery of QEEs.

Herein, we show that a stepwise-evolving artificial intelligence (se-AI) approach, based on Bayesian optimization (BO) and random forest (RF) classification can significantly shorten the development period of novel platinum-group-metal (PGM) free OER electrocatalysts with electrochemical properties comparable to those of PGM-based electrocatalysts. The concept of stepwise evolution is known as a model for primate social evolution, and it is posited as a plausible mechanism for developing highly complicated social behavior over time, leading to stable and well-functioning sociality.^12^ We drew inspiration from this model to infer evolution of society with high complexity to establish a human–machine collaboration in order to advance the development of OER electrocatalysts, which is indeed a complicated system as well. In this work, we selected 11 elements as the entities of PGM-free OER electrocatalysts to construct the model quinary-element electrocatalysts (QEEs), consisting of five different elements in the equivalent or 1:1:1:1:0.5 ratio, to build up a human–machine collaboration (Figure 1). This gives a material search space with 2772 candidates, *i.e*., a sum of _11_C_5_ = 462 (for equivalent ratio) and 5 × _11_C_5_ = 2310 (for 1:1:1:1:0.5 ratio). Therefore, about 3,000 candidates for QEEs exist. Previous computational studies on materials discovery based on Bayesian optimization suggested that the best material can be found by conducting experimental trials within 10% of the total number of candidates.^13,14^ Hence, we have established a model material search space wherein the total count of candidates approximates 3000. This total candidate number is suitable for the purpose of executing a proof-of-concept delineating human–machine collaboration in the pursuit of novel oxygen-evolving electrocatalysts while taking into account the possibility of finding the best material within 10% of experiments in whole candidates.

Although multicomponent OER electrocatalysts are of great interest due to their promising electrochemical properties,^15−20^ it is still a challenge to find out the best-performing materials because of a huge number of candidates. Furthermore, affordable OER electrocatalysts having high activity at near-neutral pH conditions are also missing. Aiming to solve these issues, in our scheme, the 10 QEEs were randomly selected as the initial data set, and 16 QEEs were synthesized by following the suggestions from BO. These QEEs resulted in a clear classification of those with better property and those with useless property. Then, we used RF to classify the candidate QEEs into two groups and performed BO for the candidates classified as having better properties. Here, our AI has achieved stepwise growth and can propose many QEEs that show small overpotentials. As the result, we were able to reach to optimized materials only after the 44 materials synthesis, by following the AI’s suggestions, indicating that only 2% of the entire pool of candidate QEE electrocatalysts was required for optimization.

## Results and Discussion

### OER Performance Optimization of Quinary-Element Electrocatalysts by Artificial Intelligence

The development of new electrocatalysts with excellent performances for the OER was proceeded by a powerful design and high-throughput calculation of AI. As shown below the OER under alkaline conditions can be described by the four reaction steps.^21,22^ To improve the properties of this complicated 4-electron-transferring electrode process, a specific combination of elements is indispensable to design a high OER activity as well as durability.

**Figure 2:**
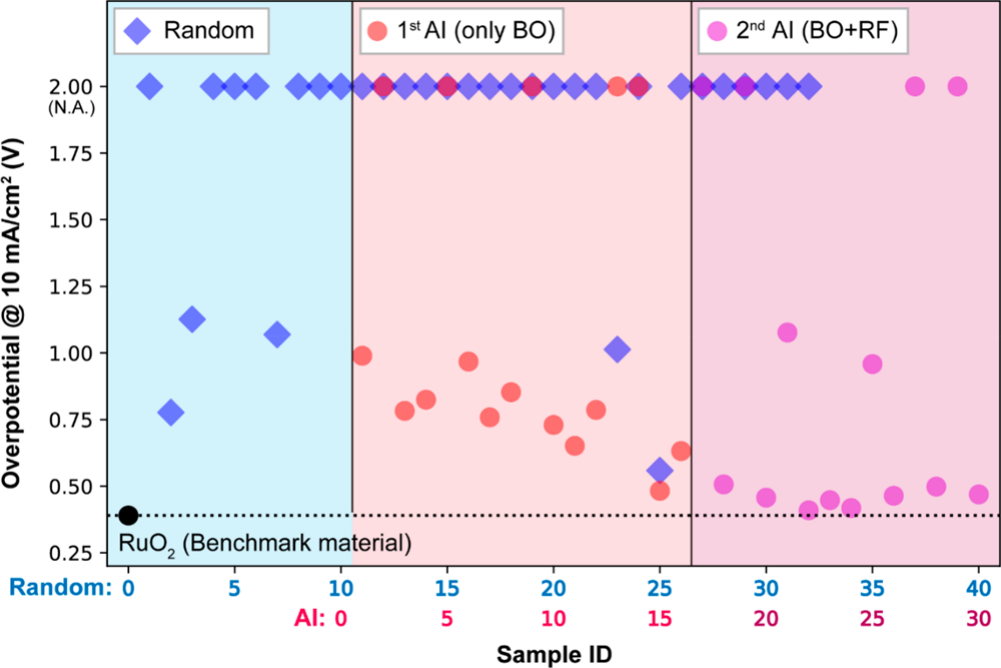
Development of OER activity along with experimental number. Overpotential at a current density of 10 mA/cm^2^ of the candidate QEE materials designed by the AI calculation (filled circles in red) compared with the candidate QEE materials from the random table (filled diamonds in blue). Red and magenta points are suggested by 1st AI and 2nd AI, respectively. The sample ID 11 is the first material suggested by AI. The minimum overpotential is obtained at the 22nd suggestion from AI.

In order to find out the best combinations of elements as quickly as possible, as we already mentioned, we selected 11 elements to synthesize the model quinary-element electrocatalysts (QEEs) with the material research space having about 3000 candidate materials (Figure 1). Based on the experimental data collected from 10 randomly designed QEE samples, it was used as a training data set for AI calculation under statistical model and optimization algorithms to predict the promising potential composition of QEEs. The AI-driven experiment is further undertaken by synthesis and electrochemical measurement tests for revealing good candidates for OER electrocatalysts, and subsequently, the OER performance, such as the activity of the prepared QEEs, is collected as an input data set for the next loop of AI calculation, as illustrated. The electrocatalytic activity of OER devoted to different elemental compositions in QEEs designed by two strategies was investigated. In the first half of the AI calculation (1st AI loop), BO using PHYSBO was performed,^14^ 16 QEEs were synthesized by selecting two QEEs in each loop, and 8 optimization loops were conducted. The 11-dimensional composition information for QEEs was used as the descriptor for AI training. For comparison, 16 materials were synthesized randomly, and BO found better materials than random selection (Figure 2), indicating that BO is a powerful tool to discover better target materials. At this time, there are 42 data sets and some of them can be classified as a smaller overpotential group and others as a higher overpotential group. Better electrocatalysts should have lower overpotentials thus, in the second half of the AI calculation, we trained the RF classifier using scikit-learn to select appropriate QEEs candidates classified as materials having smaller overpotentials and performed 6 loops of BO on these candidates. In each loop, two QEEs were selected, and the RF classifier was retrained. We note here that the metal compositions of prepared electrocatalysts are identical to the target compositions of 1:1:1:1:1 or 1:1:1:1:0.5 as confirmed by inductively coupled plasma optical emission spectrometry (ICP-OES) analysis (Table S2 and Figure S17).

The QEEs from the AI suggestion exhibited lower overpotentials at a current density of 10 mA/cm^2^ and faster reaching to better performing OER electrocatalysts comparable to ruthenium oxide (RuO_2_: the benchmark material in this study) than the QEEs from the random design (Figure 2). In particular, our second AI successfully proposed various high-performance QEEs. This result indicates that the se-AI approach is a significantly powerful method for discovering novel OER materials with decreasing time consumption in experimental processes. In the 22nd QEEs by AI suggestion, the elemental combination and atomic composition, Mn: Fe: Ni: Zn: Ag = 1:1:1:0.5:1 (AI22) showed the highest OER activity in the lowest overpotential values of the other samples until the synthesis of 48 unique materials (= 10 training data set +32 data set from first AI loop +6 data set from second AI loop). Because we found that the OER activity was saturated at this point, the investigation of further QEEs was halted and the topmost five QEEs (Table S1) were selected to investigate the origin of the high OER activity of AI22. This result indicates that we were able to reach optimized materials only after 48 materials synthesis, indicating that this approach required only 2% experimental trials of the entire candidate pool.

### Data-Driven Analysis on Discovery Process for Highly-Active Electrocatalysts with On-Demand Elements

**Figure 3:**
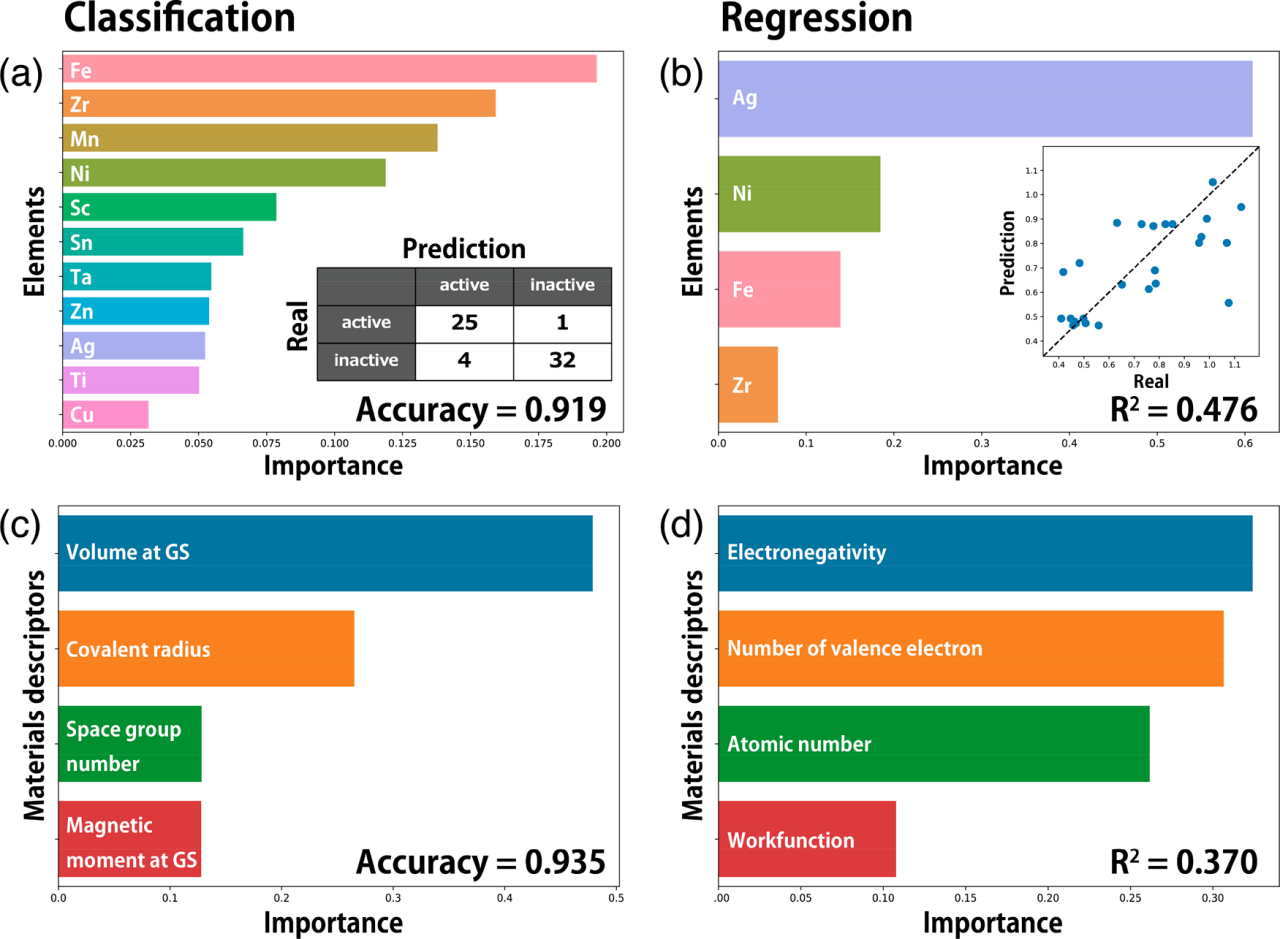
Data-driven analysis for quinary-element electrocatalysts. (a) Feature importance in RF for classification of active or inactive when the compositions of elements are used as features. The inset is the confusion matrix for 5-fold cross-validation. (b) Feature importance in RF for regression of overpotential when the compositions of elements are used as features. The inset is the scatter plot of validation data for 5-fold cross-validation. (c) Feature importance in RF for classification when the materials descriptors are used as features. (d) Feature importance in RF for regression when the materials descriptors are used as features.

Why was the se-AI approach so successful for the discovery in highly OER active QEEs? The most important factor was the accuracy of screening active compositions by the RF in the second AI loop. Including random experiments, a total of 62 experiments were performed, and the accuracy of 5-fold cross-validation was 0.919 for the material classification of active or inactive by RF. Surprisingly, this accuracy indicates that the screening of active compositions with a probability of more than 90% can proceed, and therefore the searching process for better QEEs was dramatically speeded up. The importance evaluation for each element in the RF was analyzed and the result unveiled that the compositions with Fe, Zr, and Mn have a significant effect on the classification (Figure 3a). On the contrary, the importance of Ag, Ti, and Cu was suggested as low from the view of the data-driven analysis, and therefore these elements have less effect on the material classification in active or inactive. Next, the regression performance for overpotential was also evaluated by focusing only on the active compositions of the 26 data (Figure 3b). The BO algorithm in the se-AI is based on a Gaussian process regression. However, this model was found to be unsuccessful in evaluating the importance of elements. Therefore, the regression was performed by employing RF. In the RF-based analysis, the feature selection was performed by the backward elimination strategy, because the prediction accuracy was extremely poor if the composition of all elements was used as a feature vector. The four important elements were found in the RF-based analysis. Worth mentioning, Ag being categorized as a less-important element in the classification, was placed at the top of the list (Figure 3b). This indicates that the existence of Ag is strongly related to the overpotential value among the active compositions. The top-five QEE samples contain Ag (see Table S1), and almost compositions without Ag showed poor overpotential value among active compositions. On the other hand, since the numbers of inactive and active compositions with Ag are almost the same, the presence or absence of Ag is not helpful for classification (Figure 3a). This result suggests that a selection and combination of appropriate machine-learning algorithms are critical keys for discovering target materials. Furthermore, this result also indicates that although the prediction accuracy is not high (*R*^2^ = 0.476), target materials (in the case of this study, materials with lower overpotentials) were able to be predicted by machine-learnings based only on composition of elements if right algorithms are selected. Indeed, the selection of appropriate algorithms is one of the reasons for the success of our se-AI approach leading to the discovery of unconventional OER electrocatalysts.

Further data-driven analysis can also provide an opportunity to obtain the relationship between material descriptors and OER performance. These analyses provide the mechanistic reason why the se-AI can suggest better OER catalysts so efficiently. Here, 24 types of material descriptors were prepared by only using compositional information (see Supporting Information for details). The feature selection was performed by backward elimination, and four important features were selected for classification and regression, respectively (Figure 3c,d). The volume and covalent radius are strongly effective for the classification to distinguish active and inactive materials, which classification approach was found to be an approximative estimation of OER performance. As both volume and covalent radius are correlated to elements, this result indicates that a choice of elements is loosely corresponding to OER activity. Conversely, it is revealed that parameters such as electronegativity, work functions, and the number of valence electrons, extracted through regression analysis, serve as crucial factors in achieving precise control over the overpotential value. These findings underscore the capability of our se-AI in accurately predicting the desired compound: the specially crafted AI framework exhibits the ability to replicate the key observations that form the cornerstone of modern microscopic electrochemistry theories.^23−27^

### In-Depth Electrochemical Investigations on Discovered Promising Materials

**Figure 4:**
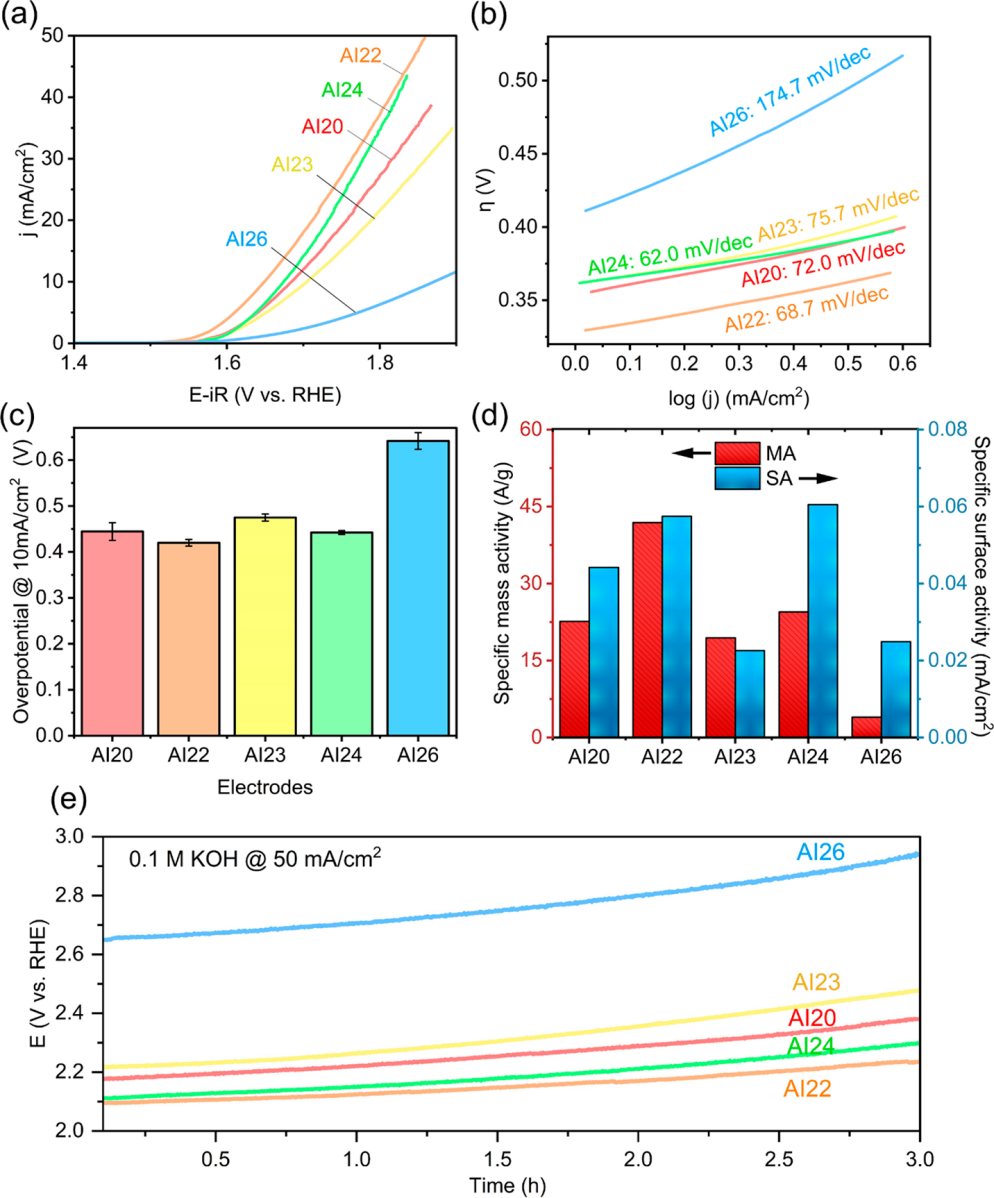
OER performances of the represetative QEE electrocatalysts in 0.1 M KOH electrolyte. (a) LSV polarization curves. (b) Tafel slopes. (c) Overpotentials at a current density of 10 mA/cm^2^ (*n* = 3). (d) Specific mass activity (MA) and specific surface activity (SA) of the QEE materials at 1.65 V vs RHE. (e) CP curves (potential vs time) measured at a current density of 50 mA/cm^2^ for 3 h in order to check the stability of materials.

The relationship between the OER performance and the elemental composition of QEEs materials was investigated to unveil a high OER performance of AI22 (also denoted as MnFeNiZn_0.5_Ag as the composition is Mn:Fe:Ni:Zn:Ag = 1:1:1:0.5:1). The electrocatalysts coated as thin-films on metallic titanium substrates were employed as a working electrode in a standard three-electrode setup with a 0.1 M KOH electrolyte (pH13). The OER activity of the AI22 was compared with other 4 AI-designed QEEs listed in Table S1: AI20 (MnFeNiZnAg), AI23 (Ti_0.5_MnFeNiAg), AI24 (MnFeNiZnAg_0.5_), and AI26 (Sc_0.5_MnFeNiAg). Comparing these QEEs consisting of Mn, Fe, Ni, Zn, and Ag, the activity of the AI22 presents the highest current densities reaching 50 mA/cm^2^ in a linear sweep voltammetry (LSV) curve than that of the AI20 and AI24 (Figure 4a). Moreover, replacing Zn with Ti (AI23) or Sc (AI26) in the QEEs significantly decreased the activity of the material. We confirmed that the addition of Ag to the QEEs was also found to be essential to trigger the OER activity for the AI22 material by comparing the electrochemical properties with the electrocatalyst without Ag (Figure S1).

The kinetic features of the AI-proposed QEE materials were evaluated from the Tafel slopes. As shown in Figure 4b, the Tafel slope of AI22 was 68.7 mV/dec, smaller than the AI20 (72.0 mV/dec), AI23 (75.7 mV/dec), and AI26 (174.7 mV/dec), but the AI24 material with a decrease in Ag ratio exhibited the lowest Tafel slope (62.0 mV/dec). This result suggests that as the Tafel slopes of the AI20, AI22, AI23, and AI24 materials are similar values, the kinetic features such as the rate-determining step of these 4 materials can be identical. However, the AI26 material shows a different Tafel slope value. This different Tafel slope in the AI26 material could be because of its high charge transfer resistances as shown later. The overpotentials at 10 mA/cm^2^ are shown in Figure 4c. The lowest overpotential was observed for the AI22 with 420 mV among other QEE materials. The electrocatalysts containing Ag with different ratios (AI20), Ti (AI23), and Zn with different ratios (AI24) respectively showed similar overpotentials of 445, 475, and 442 mV, while the presence of Sc (AI26) resulted in a high overpotential of 642 mV. It indicated that the OER activity of QEEs enhances by the existence of Mn, Fe, Ni, Zn, and Ag with the optimal elemental ratio. To determine the electrochemical active surface area (ECSA) of the QEEs, the ECSA was obtained by the roughness factor calculated by the double-layer capacitance (C_dl_) of the electrocatalysts, which was derived from the cyclic voltammogram (CV) in the non-Faradaic potential region at different scan rates (Figure S2 and methods in the Supporting Information). The highest active surface area was obtained from the AI23 material and the lowest from the AI26 material (Figure S3). The ECSA values for each material are used to calculate specific current densities based on surface area, i.e., specific surface activity (SA). The specific mass activity (MA, normalized to the mass loading of electrocatalyst) is another important feature in electrochemical properties. These SA and MA represent the key fundamental features correlated to the intrinsic activity of electrocatalysts, such as active site numbers. The MA and SA of the QEE materials were compared at a potential of 1.65 V vs RHE for OER, as presented in Figure 4d). The AI22 QEE showed the MA of 41.9 A/g and the SA of 0.057 mA/cm^2^_ECSA_, which are more than 2-fold and 1.5-fold higher than those for the other QEEs, except the AI24 material (SA = 0.061 mA/cm^2^_ECSA_). It implied excellent OER activity due to the intrinsic activity of QEEs. We further checked the stability of the AI-QEEs via chronopotentiometry (CP) measurement at 50 mA/cm^2^ for 3 h in 0.1 M KOH (Figure 4e).

The above results show that AI22 is the best OER electrocatalyst in the present material research space. Therefore, a wide spectrum of physical characterizations was applied to the AI22 material. The AI22 material (MnFeNiZn_0.5_Ag) was determined to consist of AgCl nanoparticles dispersed within an amorphous matrix based on oxychloride, containing Mn, Fe, Ni, Zn, O, and Cl elements. Other highly active electrocatalysts are also materials based on AgCl with different compositions of amorphous oxychloride matrixes. For a comprehensive understanding of these materials, detailed characterizations including scanning electron microscopy, X-ray photoemission spectrometry, and high-resolution transmission electron microscopy can be found in the Supporting Information: Figures S4–S19, Tables S2–S4, and Supporting Discussions S1 and S2. Although the precise mechanism behind the superior activity of the AI22 material remains unclear and falls beyond the scope of this study, these observations strongly suggest that a synergistic effect akin to a heterojunction plays a pivotal role in both the activity and stability of the material:^28,29^ the heterojunction material, composed of AgCl and an amorphous oxychloride matrix containing multiple metals, serves to enhance the OER activity, and also this effect could be a reason for the good stability of the materials.

### OER Performance of the AI22 material (MnFeNiZn_0.5_Ag) in Near-Neutral Electrolytes

**Figure 5:**
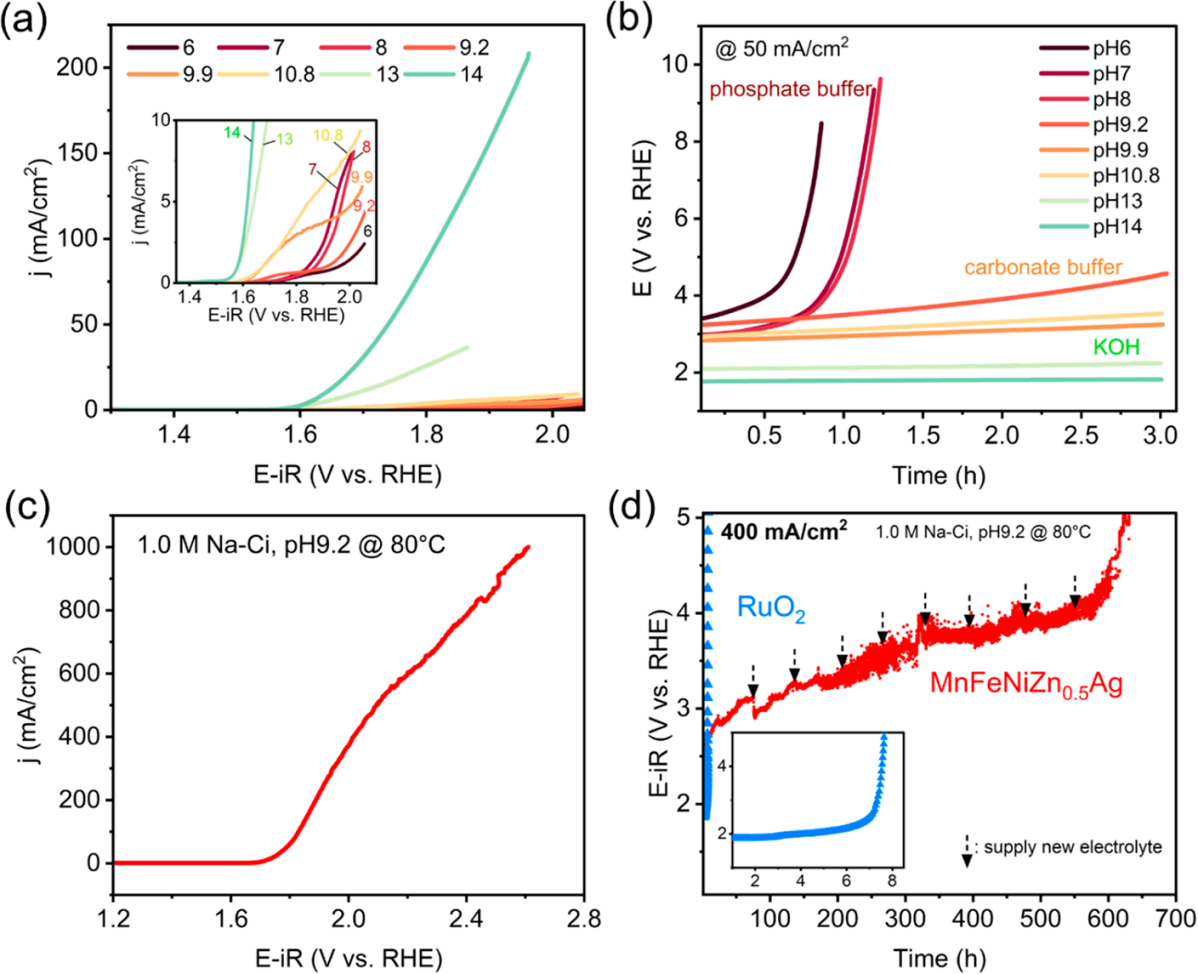
OER performance of the AI22 material (MnFeNiZn_0.5_Ag). (a) LSV curves and (b) chronopotentiometry (CP) curves at a current density of 50 mA/cm^2^ measured at room temperature (25 °C) in different pH regions of electrolytes: pH 6, 7, and 8 (0.1 M K-Pi), pH 9.2, 9.9, and 10.8 (0.1 M Na-Ci), pH 13 (0.1 M KOH), and pH 14 (1 M KOH). The OER performances at high temperature (80 °C) in 1.0 M Na-Ci electrolyte (pH 9.2): (c) LSV curve of the AI22 material and (d) CP curves of the AI22 material compared with the RuO_2_ electrode (inset) at a high current density of 400 mA/cm^2^.

The OER activity and stability of the best material of the AI22 material (MnFeNiZn_0.5_Ag) were further studied in neutral to mild alkaline electrolyte conditions to demonstrate its promising electrochemical property. First, we focused on the activity by checking the current densities. In the high pH condition (1 M KOH, pH 14), the current density of the AI22 material easily reaches 200 mA/cm^2^ at 1.95 V vs RHE (Figure 5a). This result is obvious because of the presence of a high amount of hydroxide ion (OH−) leading to kinetically favorable for OER.^30,31^ In contrast, the OER activities of the AI22 material in 0.1 M phosphate (K-Pi) and carbonate (Na-Ci) buffer electrolytes under neutral pH and mild alkaline conditions exhibited low current densities (<10 mA/cm^2^).

Next, we focused on durability by checking the change of reaction potential at 50 mA/cm^2^ for 3 h. Under the pH13–14 conditions, the AI22 material is stable (Figure 5b). Furthermore, even if the pH was decreased to near-neutral regions (0.1 Na-Ci, pH 9.2–10.8), the AI22 material was still stable. All elements in the AI22 material were homogeneously distributed and also the composition remained after the stability test in alkaline and near-neutral electrolytes (Figures S18 and S19). However, at lower pH < 8 in the phosphate electrolytes, the potential rapidly increased within 1 h. This result suggests that the material was degraded during the OER because transition metals such as Ni, Fe, and Mn were dissolved in neutral or acidic environments.^32−34^

Based on the above results, we further investigated the OER performance of the AI22 material at a high temperature of 80 °C in 0.1 M Na-Ci and 1 M Na-Ci (pH 9.2) in comparison with the benchmark RuO_2_ electrode. It is noted that the elevated temperature provides more kinetically favorable condition for the AI22 material in the high concentration of buffer (1 M Na-Ci). We confirmed that the higher OER activity of the AI22 material was obtained at the 1 M Na-Ci compared to the low concentration of 0.1 M Na-Ci (Figure S20). In the 1 M Na-Ci electrolyte, the OER current density of the AI22 material reached 1000 mA/cm^2^. Furthermore, this material is stable for over 600 h at 400 mA/cm^2^ (Figures 5c,d). Although the initial activity of RuO_2_ was slightly higher than the AI22 material (Figure S21), RuO_2_ is stable for only 7 h at 400 mA/cm^2^. This observation confirms the outstanding stability of the AI22 materials compared to RuO_2_, which is suffered by the dissolution and deactivation during the OER.^35−38^ Thus, the AI22 material exhibits both high activity and superior stability compared to the benchmark PGM-based electrocatalyst of RuO_2_.

**Figure 6:**
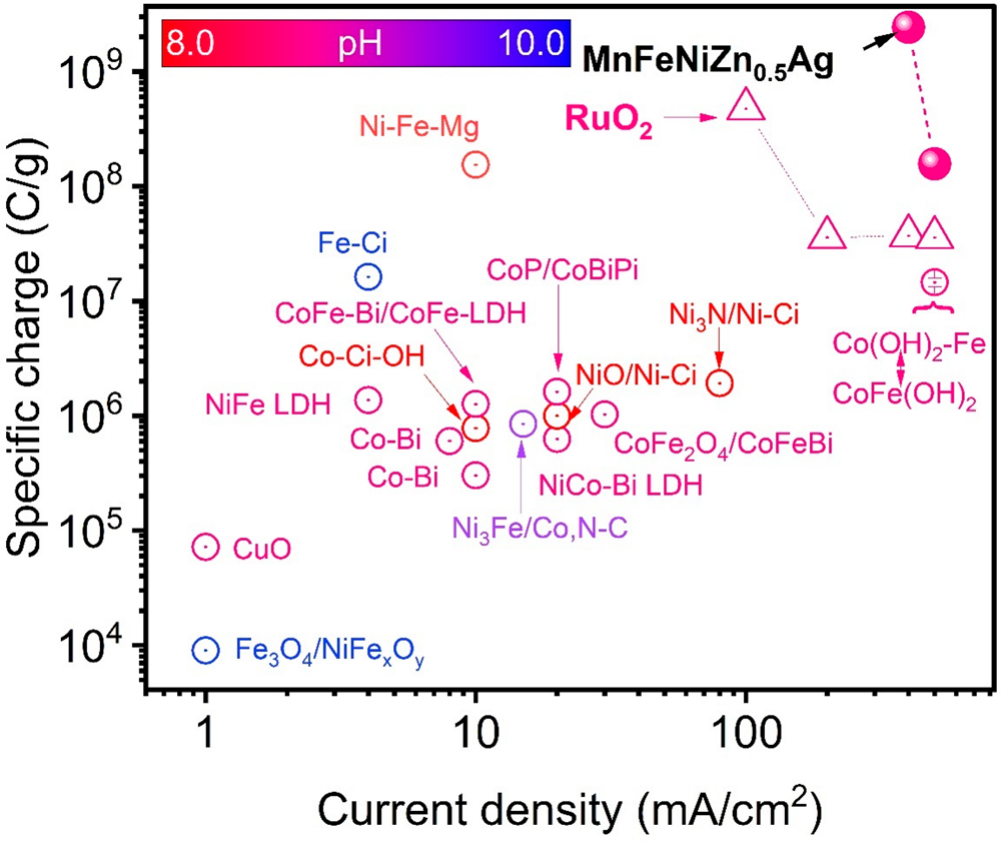
Ragone plot (the specific charge (C/g) vs current density) of the AI22 material (MnFeNiZn_0.5_Ag), comparing with the values of representative affordable OER electrocatalysts. The previously reported as well as a RuO_2_ benchmark materials in pH-near-neutral electrolytes are shown in this figure. pH 8.3: Co-Ci–OH,^39^ Ni_3_N/Ni-Ci,^40^ NiO/Ni-Ci.^41^ pH 8.5: Ni–Fe–Mg.^42^ pH 9.2: MnFeNiZn_0.5_Ag (80 °C), RuO_2_ (80 °C), NiCo-Bi-LDH,^43^ Co–Bi,^44,45^ CoFe_2_O_4_/CoFeBi,^46^ NiFe LDH,^47,48^ CuO,^49^ CoP/CoBiPi,^50^ CoFe-Bi/CoFe LDH,^51^ Co(OH)_2_-Fe to CoFe(OH)_2_ (80 °C);^52^ pH 9.45: Ni_3_Fe/Co,N–C.^53^ pH 9.75: Fe-Ci,^54^ Fe_3_O_4_/NiFe_*x*_O_*y*_.^55^

The Ragone plot based on a specific charge was prepared to illustrate the extraordinary electrochemical property of the AI22 material under near-neutral electrolytes (pH 8 to 10) by comparing other reports. The plot indicates the superior electrochemical properties of the AI22 material not only compared to other affordable electrocatalysts but also to the PGM-based material of RuO_2_ (Figure 6 and Table S4).

## Conclusion

In this study, we have successfully discovered high-performance OER electrocatalysts based on the QEE materials by using AI to accelerate human experiments. The AI-suggested QEE composed of Mn, Fe, Ni, Zn, and Ag (denoted as AI22) demonstrated exceptional OER activity, resulting from the synergistic effect produced by the unique elemental combination and morphology. We also checked the AI22 material as an OER electrocatalyst in near-neutral electrolytes at 80 °C: this material demonstrated excellent activity, reaching 1000 mA/cm^2^, and outstanding durability at a high current density of 400 mA/cm^2^ for over 600 h in 1.0 M Na-Ci (pH 9.2). As a result, this material exhibited a higher specific charge than that of RuO_2_. Consequently, this study demonstrates that human–machine collaboration with se-AI is a promising approach for accelerated discovery of high-performance unconventional electrochemical materials with affordable elements having the potential for replacing conventional standard materials based on PGMs. Moreover, this study represents a foundational model study, devised to exemplify the concept of human–machine collaboration in the novel materials discovery, employing the OER electrocatalyst as an example system. Therefore, a myriad of alternative parameters are available for further material design. These parameters encompass key strategies for materials design such as alloying or demixing structures, facilitating surface texture and morphology controls. Thus, the material system can be extended in a multiphase system with a wide spectrum of particle sizes and compositions affecting the nonoptimized synthetic conditions in this study. These prospects on materials design hold promise, not only in the pursuit of OER electrocatalysts demonstrating superior performance to AI22, as elucidated in this study but also for dramatically enhancing material’s properties for other diverse applications. These applications may include rechargeable batteries, fuel cells, or electrochemical high-value chemical synthesis, but are not limited only to electrochemical devices. Consequently, it is our aspiration that this work could encourage scientists and engineers to harness the power of AI in material discovery, transcending the limitations of current human capabilities and thereby accelerating science and technology in the 21st century.

## Methods

The details on the experiments can be found in the Supporting Information. Here the most essential experimental procedures are described.

### Material Synthesis

For the typical material preparation, the MnFeNiZn_0.5_Ag (AI22) QEE was synthesized via a thermal decomposition process. A mixture of five metal solutions (0.05 M) was prepared by mixing stock solutions at a ratio of MnCl_2_:FeCl_2_:NiCl_2_:ZnCl_2_:AgNO_3_ = 1:1:1:0.5:1. A 2.5 μL of the mixture solution was dropped onto the Ti-substrate and dried at room temperature. Afterward, the sample was precalcined at 350 °C for 10 min in the furnace. The solution drop-cast and precalcination steps were repeated nine times, and then the sample was finally calcined at 450 °C for 1 h to obtain the metal oxide layers coated on the Ti substrate for application as electrodes.

### Electrochemistry

The electrochemical measurements of the QEEs toward OER were carried out at room temperature using a multichannel potentio/galvanostat (VMP-3, Bio-Logic, France) with a standard three-electrode configuration. The as-prepared QEE/Ti samples (geometrical surface area ∼ 0.5 × 1.0 cm^2^) were used as the working electrode with a platinum wire as the counter electrode and an Ag/AgCl electrode as the reference electrode. The electrochemical data were analyzed by EC-lab software (Bio-Logic).

### Data-Driven Approaches

In the se-AI approach, Bayesian optimization was performed by PHYSBO, and the random forest classifier was performed by scikit-learn. The feature importance in the random forest classifier and regression was also extracted by using scikit-learn. The magpie descriptor^56^ from material composition and Matminer^57^ were partially used to generate several material descriptors.

## Supporting Information Available

Detailed experimental methods, supporting electrochemical data, SEM and TEM images with elemental analysis, XPS data, supporting discussions, and supporting references (PDF)
