# Supplementary material for: Human–Machine Collaboration for Accelerated Discovery of Promising Oxygen Evolution Electrocatalysts with On-Demand Elements
Source: ACS Cent Sci. 2023 Nov 30;9(12):2216–24. doi: 10.1021/acscentsci.3c01009 (PMC10755732; doi:10.1021/acscentsci.3c01009)
Supplement: Supplementary file 1 — oc3c01009_si_001.pdf [file oc3c01009_si_001.pdf]

Supporting Information

Human-Machine Collaboration for Accelerated Discovery of Promising Oxygen Evolution Electrocatalysts with On-Demand Elements

Ken Sakaushi[1,*], *Watcharaporn Hoisang*,[1] *and Ryo Tamura*[2,3,*]

[1]Research Center for Energy and Environmental Materials, National Institute for Materials Science, 1-1 Namiki, Tsukuba, Ibaraki 305-0044, Japan.

E-mail: sakaushi.ken@nims.go.jp

[2]Center for Basic Research on Materials, National Institute for Materials Science, 1-1 Namiki, Tsukuba, Ibaraki 305-0044, Japan.

E-mail: tamura.ryo@nims.go.jp

[3]Graduate School of Frontier Sciences, The University of Tokyo, Kashiwa 277-8561, Japan.

Methods

Chemicals and materials

Manganese chloride ($MnCl_2$, 99.0%), iron (II) chloride ($FeCl_2$, 99.0%), nickel chloride ($NiCl_2$, 98.0%), silver nitrate ($AgNO_3$, 99.8%), sodium carbonate (Na_2CO_3, 99.8%) and sodium bicarbonate ($NaHCO_3$, 99.5%) were purchased from Wako Fuji Co. Ltd. Scandium chloride ($ScCl_3$, 99.0%) was purchased from Santa Cruz Biotechnology and zinc chloride ($ZnCl_2$, 99.95%) was purchase from Alfa Aesar. Titanium (IV) fluorid1e (TiF_4, 99.0%), ruthenium chloride ($RuCl_3$, 99.9%), potassium hydroxide (KOH, 99%), and oxalic acid ($H_2C_2O_4$, 99%) were purchased from Sigma-Aldrich. Ethanol (99%) and acetone (99%) were purchased from TCI. All organic solvents were degassed with nitrogen gas for an hour before use. A stock solution (0.1 M) of each metal salt was prepared in ethanol. The deionized water (Milli-Q IQ 7000; 18 MΩ cm, total organic content > 5 ppb, Merck AG, Germany) was used in the buffer electrolyte preparation. Phosphate buffers (0.1 M K-Pi, pH 6.0, 7.0, and 8.0) were purchased from Wako Fuji Co. Ltd. Carbonate buffers (0.1 M Na-Ci: pH 9.2, 9.9, and 10.8, and 1.0 M Na-Ci, pH 9.2) were prepared by mixing appropriate proportions of Na_2CO_3 and $NaHCO_3$ solution. Titanium foil (Ti, 99.5%, Nilaco) with a size of 0.05×0.5×30 mm was cleaned with acetone, etched in the $H_2C_2O_4$ solution (10% w/v) at 80°C for 1 h, and washed with deionized water to clean the substrate surface.

Materials synthesis

For the typical material preparation, the $MnFeNiZn_{0.5}Ag$ (AI22) QEE was synthesized *via* a thermal decomposition process. A mixture of five metal solutions (0.05 M) was prepared by mixing stock solutions at a ratio of $MnCl_2$: $FeCl_2$: $NiCl_2$: $ZnCl_2$: $AgNO_3$ = 1: 1: 1: 0.5: 1. A 2.5 μL of the mixture solution was dropped onto the Ti-substrate and dried at room temperature.

Afterward, the sample was pre-calcined at 350℃ for 10 min in the furnace. The solution drop-cast and pre-calcination steps were repeated nine times and then the sample was finally calcined at 450℃ for 1 h to obtain the metal oxide layers coated on the Ti substrate for application as electrodes. For the benchmark material, the mixture of the five metals solution was replaced with the 0.05 M $RuCl_3$ and the RuO_2/Ti electrode was prepared as same as the abovementioned procedures. The mass loading on the Ti substrate of all electrocatalysts was ca. 0.13 mg.

Electrochemical measurements

The electrochemical measurements of the QEEs toward OER were carried out at room temperature using a multi-channel potentio/galvanostat (VMP-3, Bio-Logic, France) with a standard three-electrode configuration. The as-prepared QEE/Ti samples (geometrical surface area ~ 0.5×1.0 cm^2) were used as the working electrode with a platinum wire as the counter electrode and an Ag/AgCl electrode as the reference electrode. The electrochemical data were analyzed by EC-lab software (Bio-Logic). All measured potentials were converted to the reversible hydrogen electrode (E vs RHE) scale, according to the Nernst equation:

$$E_{RHE} = E_{Ag/AgCl} + 0.208 + 0.059*pH$$

The OER electrocatalytic activity of electrodes was examined by linear sweep voltammetry (LSV) with a scan rate of 1 mV/s in 0.1 M KOH electrolyte. Electrochemical impedance spectroscopy analysis (EIS) was performed at the frequency range of 100 mHz to 1 MHz. The electrochemically active surface area (ECSA) of the electrocatalysts was investigated using cyclic voltammetry (CV) measurement within a non-Faradaic region of 0.6 to 0.8 V vs RHE at a different scan rate of 5, 10,

20, 30, and 40 mV/s for estimating the double-layer capacitance (C_{dl}). The ECSA value can be calculated by the specific capacitance of the sample as shown in eq 1.

$$ECSA = \frac{C_{dl}}{C_s} \qquad (1)$$

Where the general specific capacitance (C_s) is of 0.040 mF/cm^2.[S1] Then, it is divided by the geometrical surface area of the electrode (0.5 cm^2), as a roughness factor (RF).[1] The stability of electrocatalysts was carried out at a current density of 50 mA/cm^2 in 0.1 M KOH by a chronopotentiometry measurement with *iR* compensation. Specific mass activity (MA) values were calculated from the measured current (*i*) at a potential of 1.65 V vs RHE and mass loading (*m*) of the electrocatalyst as the following equation: MA = *i*/*m*. Specific surface activity (SA) was calculated by dividing the measured current (*i*) by the ECSA estimated from the RF value at a given potential. The measurement of the electrochemical performances of the $MnFeNiZn_{0.5}Ag$ QEE at high temperature (80 °C) was carried out in 0.1 M Na-Ci and 1.0 M Na-Ci electrolytes (pH 9.2). The stability performances under these near-neutral electrolytes were investigated at current densities of 50, 100, 200, 400, and 500 mA/cm^2.

Material Characterizations

Scanning electron microscopy (SEM) images were obtained using a field emission scanning electron microscope (FE-SEM; SU8000, Hitachi, Japan) operated at 15.0 kV in in the high-vacuum mode and complemented by elemental analysis using an energy-dispersive X-ray spectrometry (EDX; Quantax FQ5060, Bruker, Japan). Transmission electron microscopy (TEM) images were obtained on a 200 kV field emission transmission electron microscope (JEM-2100F1, JEOL,

Japan) or an atomic resolution analytical electron microscope (JEM-ARM200F-B, JEOL) with a Cs corrector to obtain microscopic images and energy-loss near-edge structures (ELNES) of the catalysts. The ELNES data was obtained on an aberration-corrected scanning transmission electron microscope (STEM) mode. X-ray diffraction (XRD) measurement were performed using a MiniFlex X-ray diffractometer (Rigaku, Japan) with a Cu Kα radiation (0.154 nm) with a scanning rate of 4°/min and were analyzed by SmartLab Studio II software (Rigaku). X-ray photoelectron spectrometry (XPS) was performed on a X-ray photoelectron spectrometer instrument (Quantera SXM: Ulvac-PHI, Albac Fay, Japan) equipped with a monochromatic Al Kα sources. The C1s peak at 284.8 eV was used for calibration of the binding energies. The elemental composition of the QEEs was determined by an inductively coupled plasma optical emission spectrometer (ICP-OES; SPS3500DD, Hitachi, Japan).

Detail of material descriptors

In this study, 24 types of material descriptors were used. Among them, 22 types of descriptors were generated by matminer package as magpie compositional descriptor. In the type of compositional descriptor, the fundamental values of each element were averaged by composition. Here, the following fundamental values that can be obtained in the periodic table and DFT calculations were used: atomic number, Mendeleev number, atomic weight, melting temperature, periodic table column, periodic table row, covalent radius, electronegativity, number of valence electrons, filled s orbitals, filled p orbitals, filled d orbitals, filled f orbitals, number of unfilled valence orbitals,

unfilled s orbitals, unfilled p orbitals, unfilled d orbitals, unfilled f orbitals, volume at ground state, band gap at ground state, magnetic moment at ground state, and space group number. The remaining two descriptors are work function and standard redox potential. These values were collected from Electrochemical Measurement Manual (Ed. The Electrochemical Society of Japan, 2002).

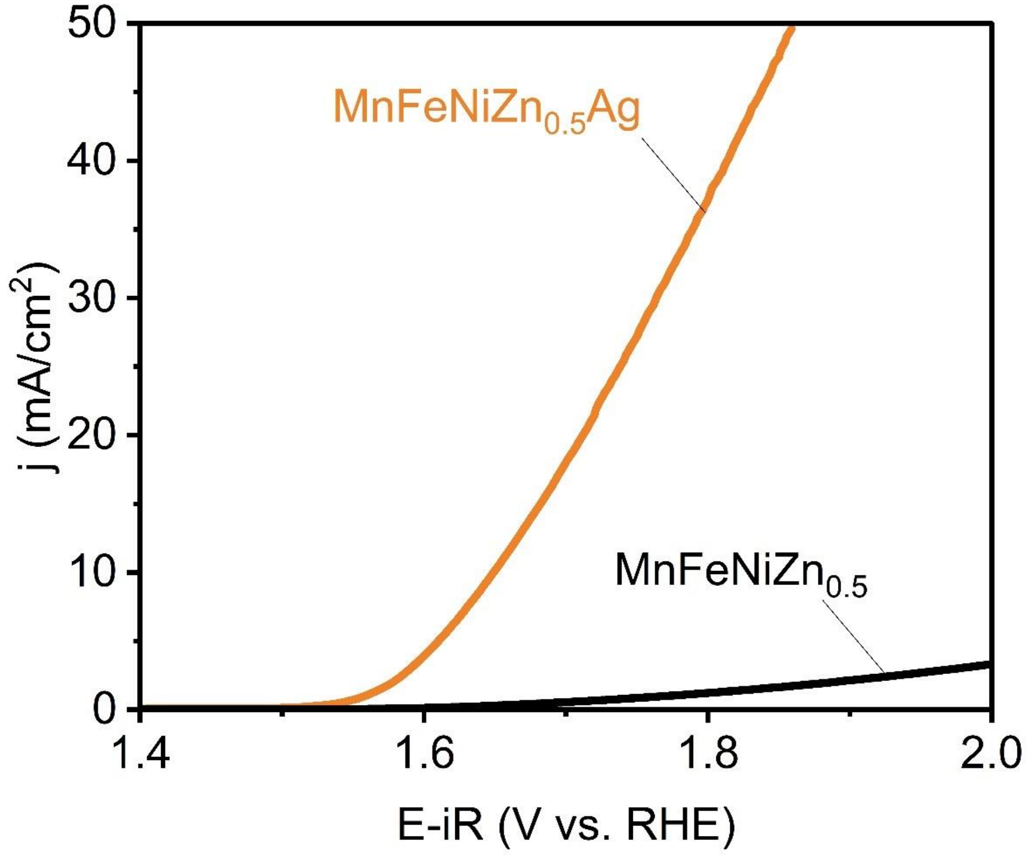

Figure S1. LSV curves of MnFeNiZn$_{0.5}$Ag (AI22) and MnFeNiZn$_{0.5}$ QEEs in 0.1 M KOH at a scan rate of 1 mV/sec.

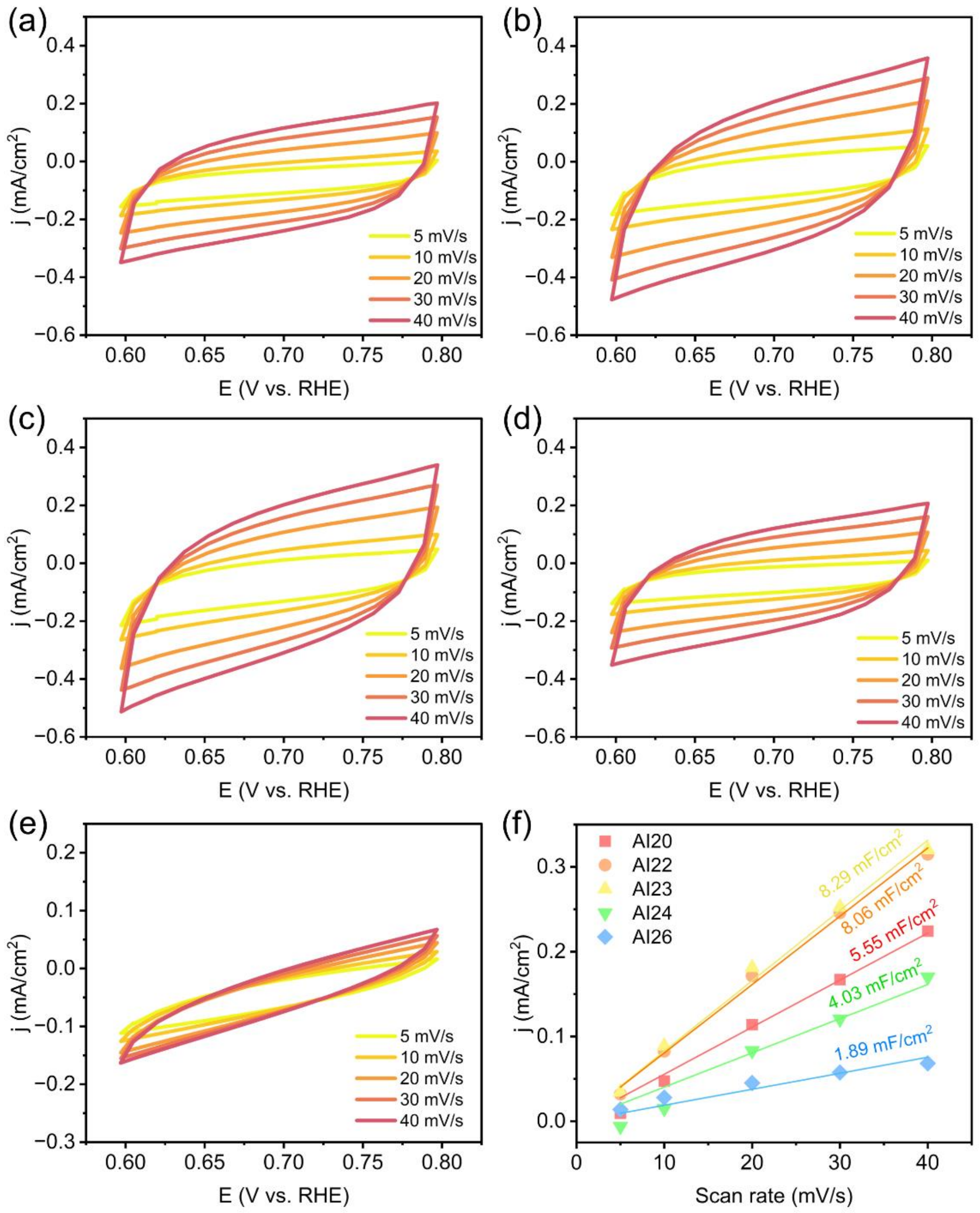

Figure S2. The double-layer capacitance curves of the QEEs; (a) MnFeNiZnAg (AI20), (b) $MnFeNiZn_{0.5}Ag$ (AI22), (c) $Ti_{0.5}MnFeNiAg$ (AI23), (d) $MnFeNiZnAg_{0.5}$ (AI24), and (e) $Sc_{0.5}MnFeNiZnAg$ (AI26). (f) Plot of current density versus scan rate and corresponding linear fitting with labeled slope values.

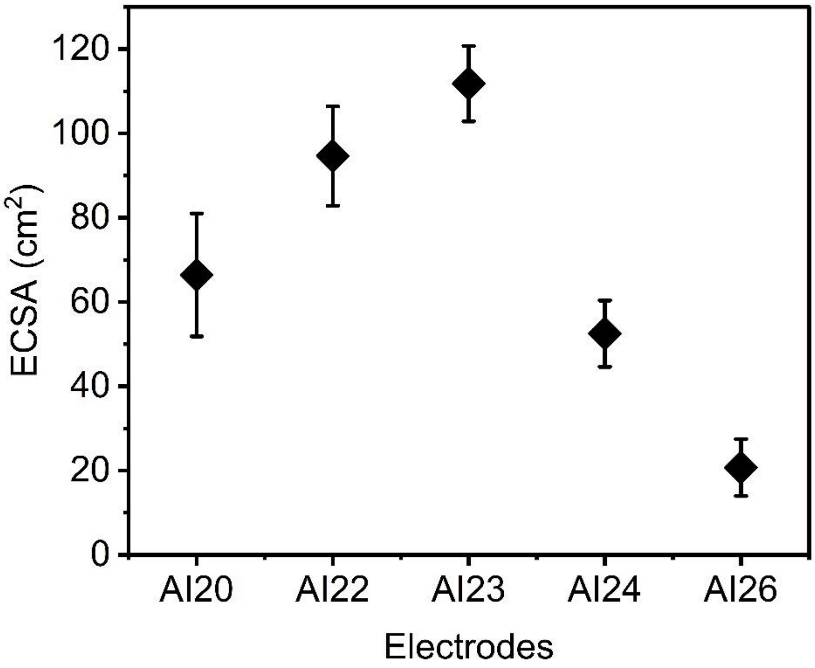

Figure S3. Average ECSA (n = 3) calculated from the RF of the QEE samples. The error was obtained by measuring independent three electrochemical experiments to calculate ECSA.

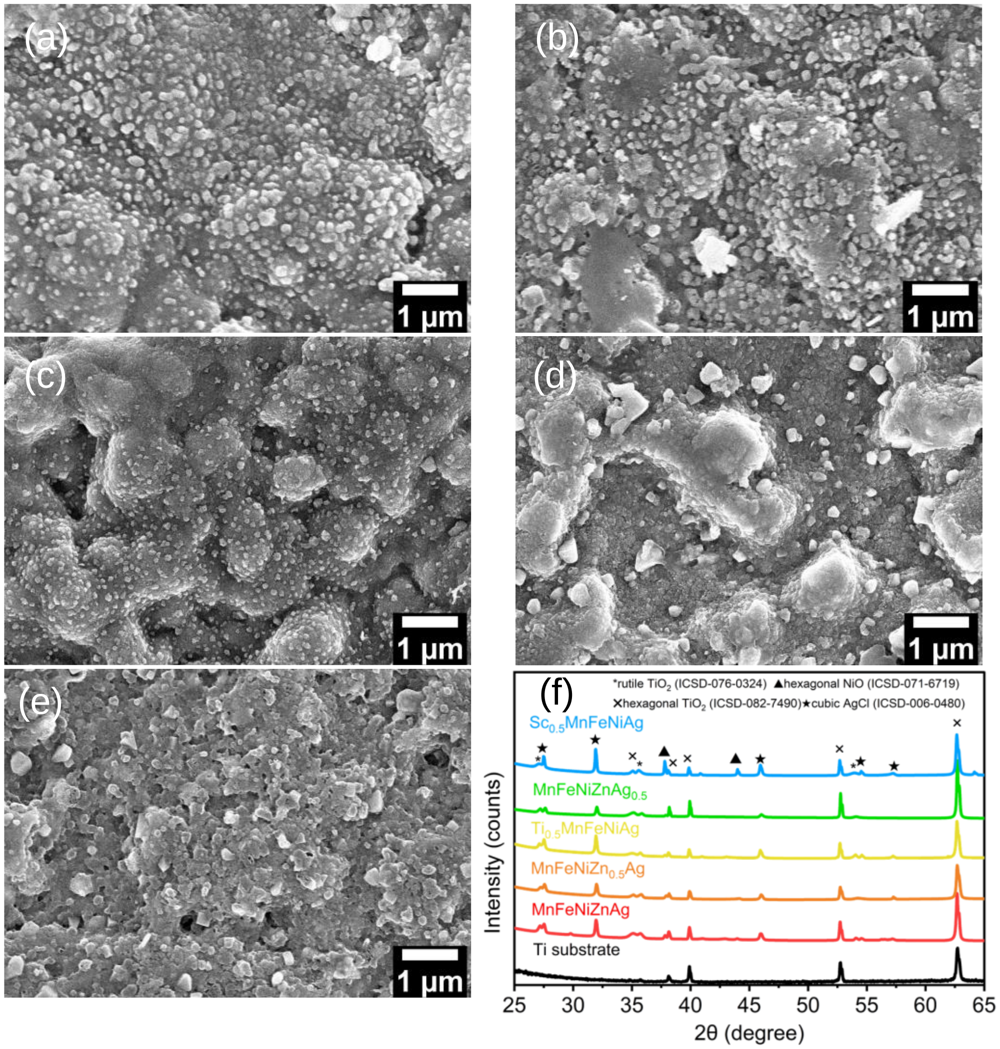

Figure S4. SEM images of QEEs for (a) MnFeNiZnAg (AI20), (b) $MnFeNiZn_{0.5}Ag$ (AI22), (c) $Ti_{0.5}MnFeNiAgO_x$ (AI23), (d) $MnFeNiZnAg_{0.5}O_x$ (AI24), and (e) $Sc_{0.5}MnFeNiAgO_x$ (AI26). (f) XRD patterns of all QEE materials. The peaks corresponding to metallic Ti and rutile TiO_2 comes from substrate.

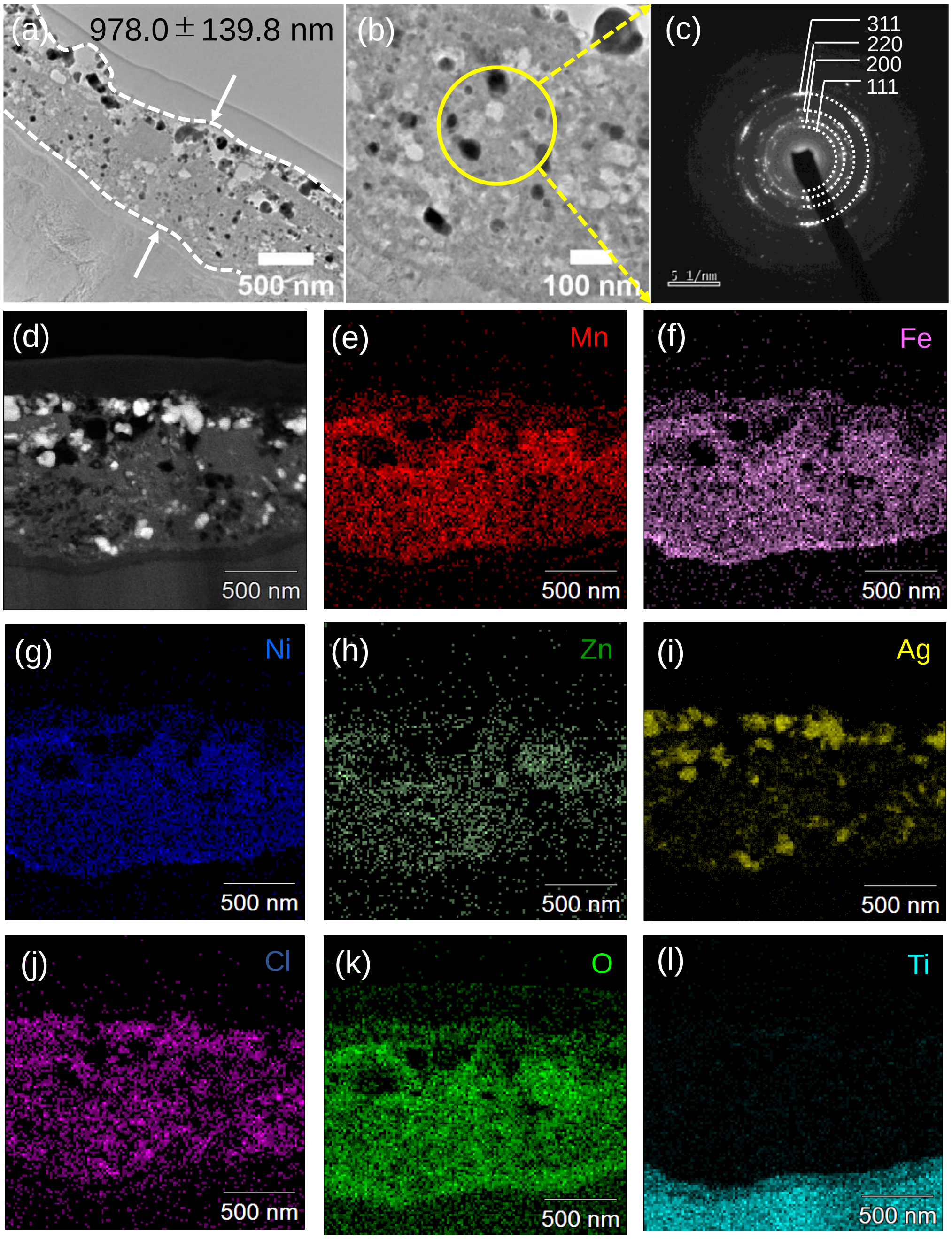

Figure S5. Morphological characterization of the AI22 material ($MnFeNiZn_{0.5}Ag$). (a) Low magnification TEM image. (b) High magnification TEM image with the yellow circle of the electron diffraction spot and (c) the corresponding SAED pattern. (d) ADF-STEM image and (e-l)

the corresponding elemental EDS mapping.

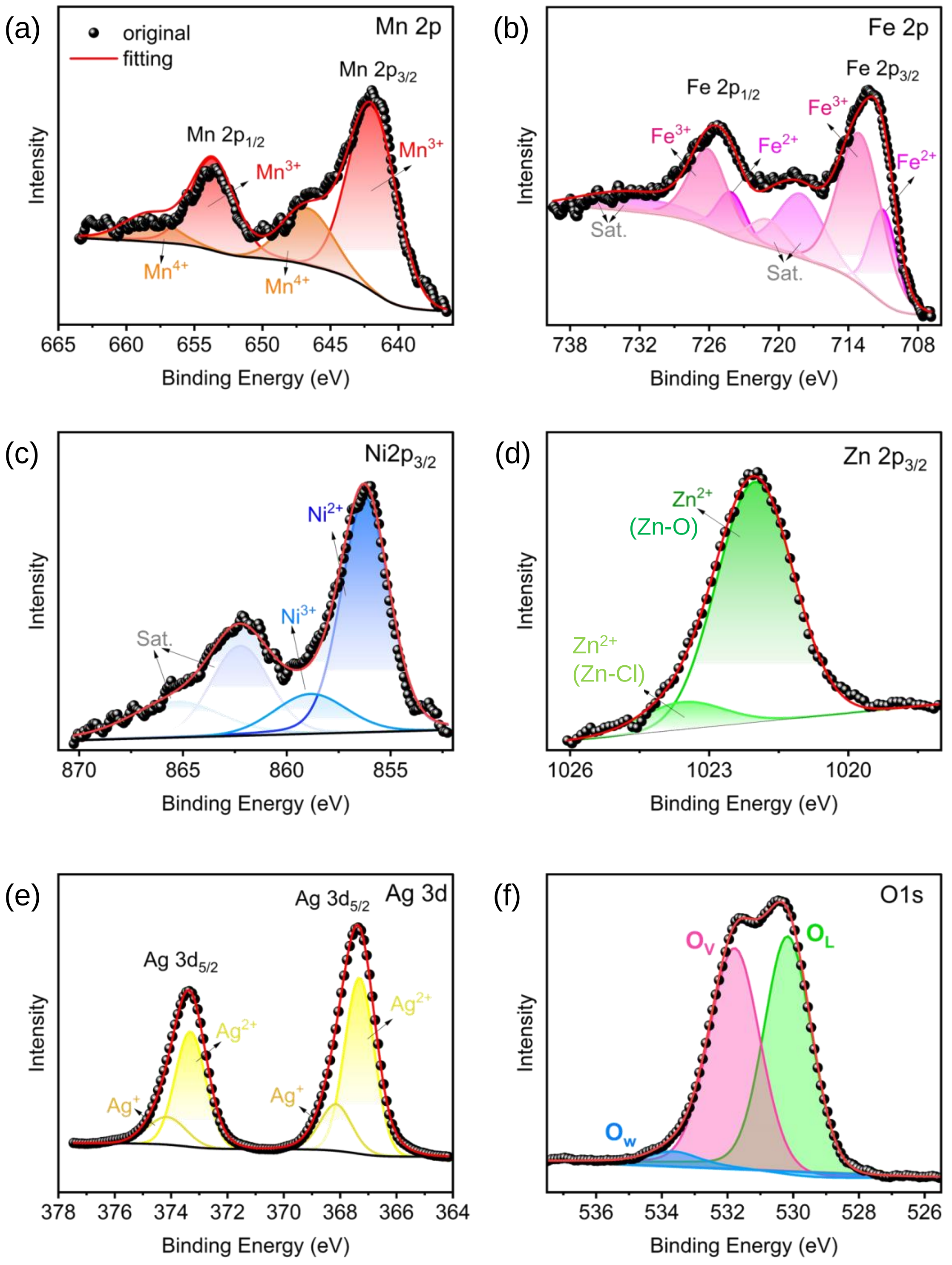

Figure S6. XPS spectra of MnFeNiZn0.5Ag (AI22) QEEs. (a) Mn 2p, (b) Fe 2p, (c) Ni $2p_{3/2}$, (d) Zn $2p_{3/2}$, (e) Ag 3d, and (f) O 1s.

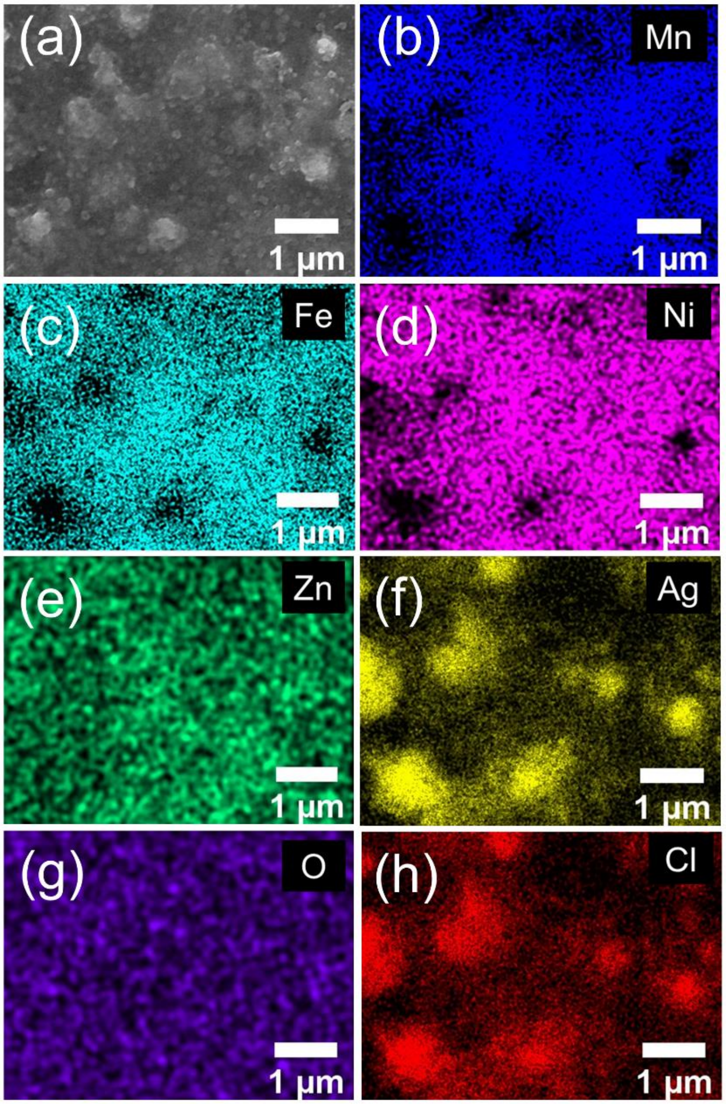

Figure S7. (a) SEM image and (b-h) the corresponding EDX mapping of the $MnFeNiZn_{0.5}Ag$ (AI22) sample.

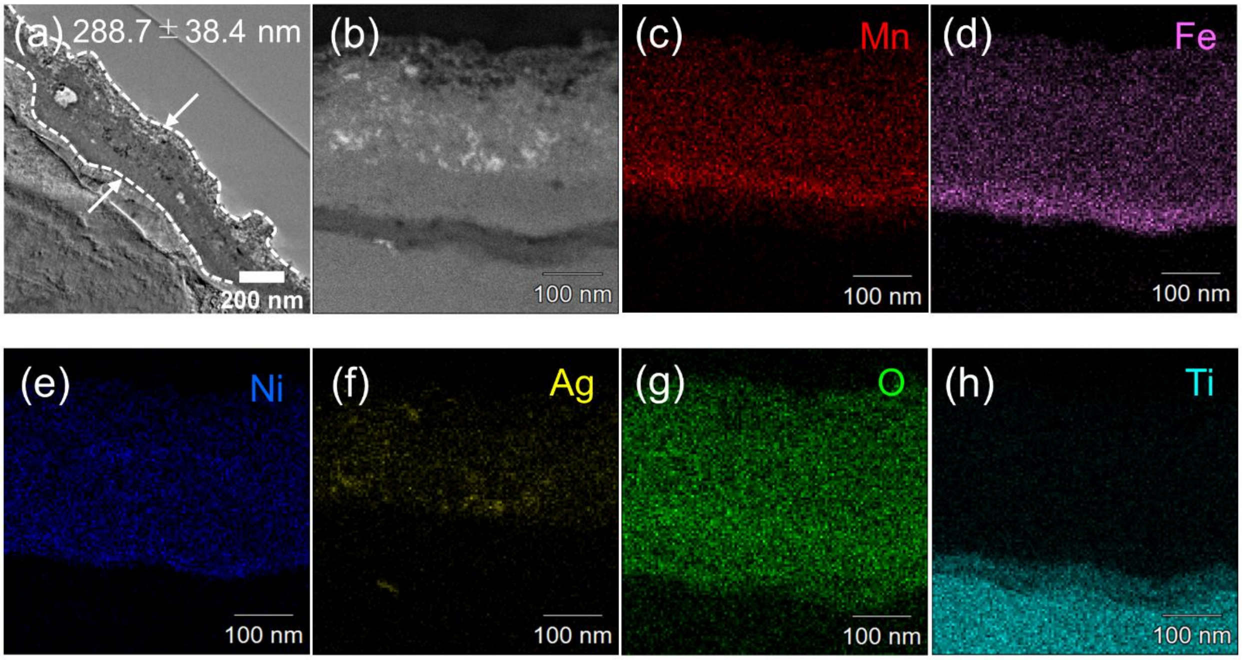

Figure S8. (a) TEM, (b) ADF-STEM images, and (c-h) the corresponding elemental EDS mapping of the $Ti_{0.5}MnFeNiAg$ (AI23) sample.

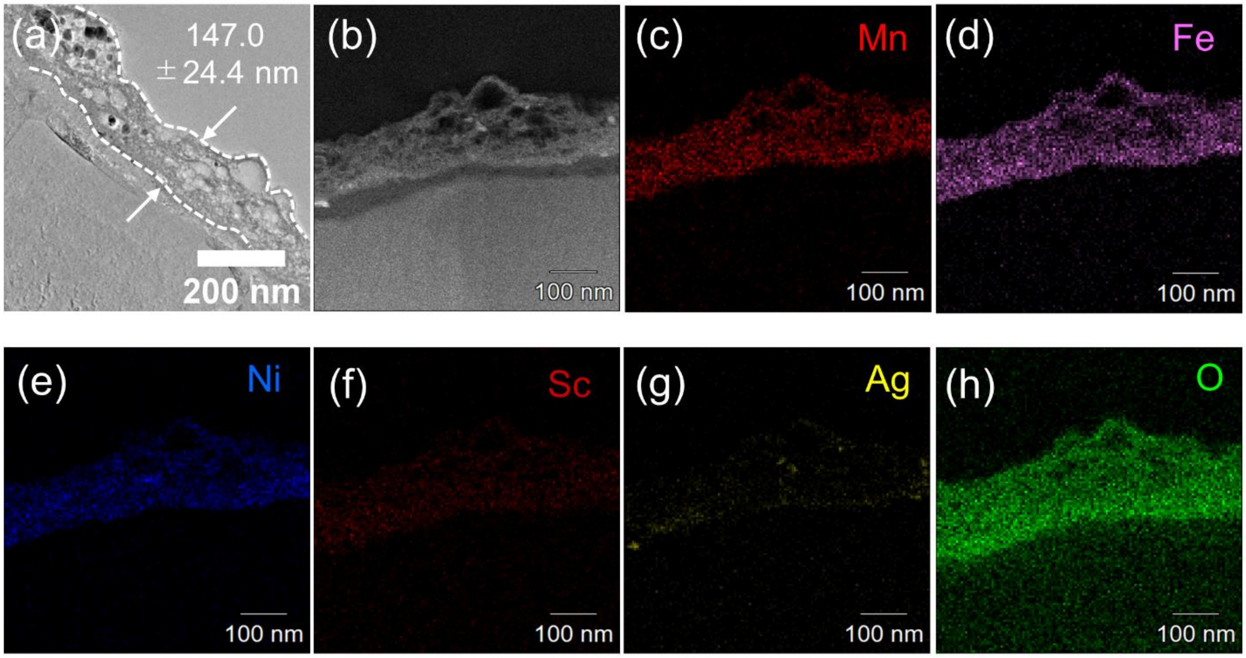

Figure S9. (a) TEM, (b) ADF-STEM images, and (c-h) the corresponding elemental EDS mapping of the $Sc_{0.5}MnFeNiAg$ (AI26) sample.

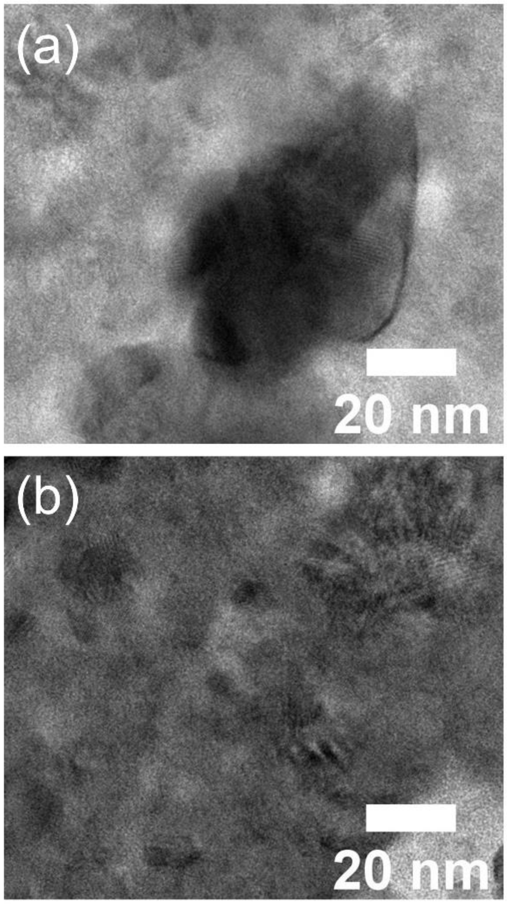

Figure S10. (a, and b) High magnification TEM images of $MnFeNiZn_{0.5}Ag$ (AI22).

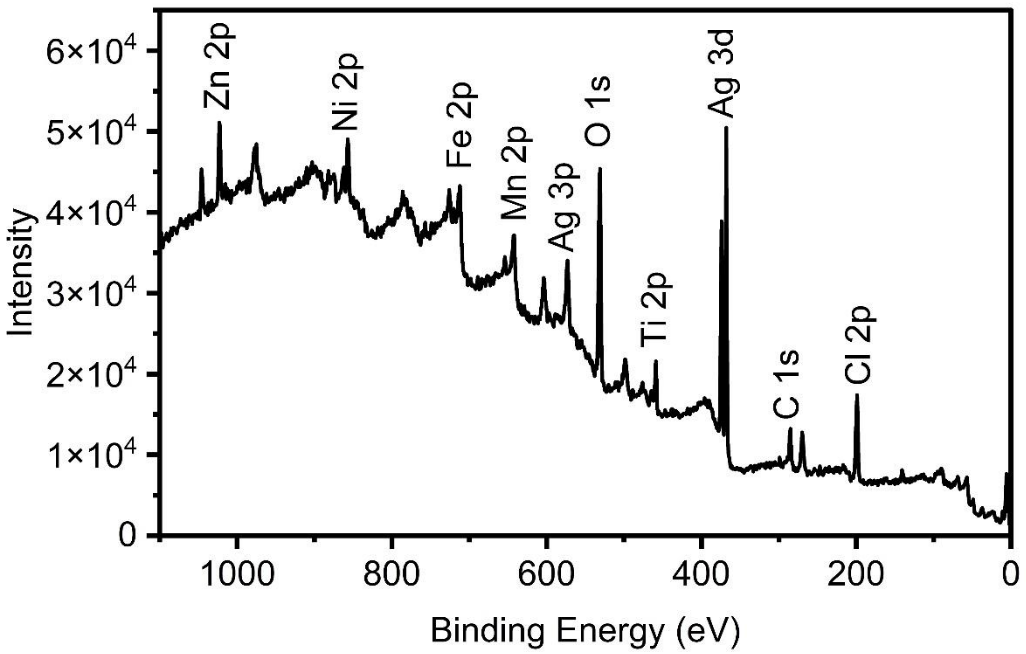

Figure S11. The full XPS spectrum of $MnFeNiZn_{0.5}Ag$ (AI22) QEE.

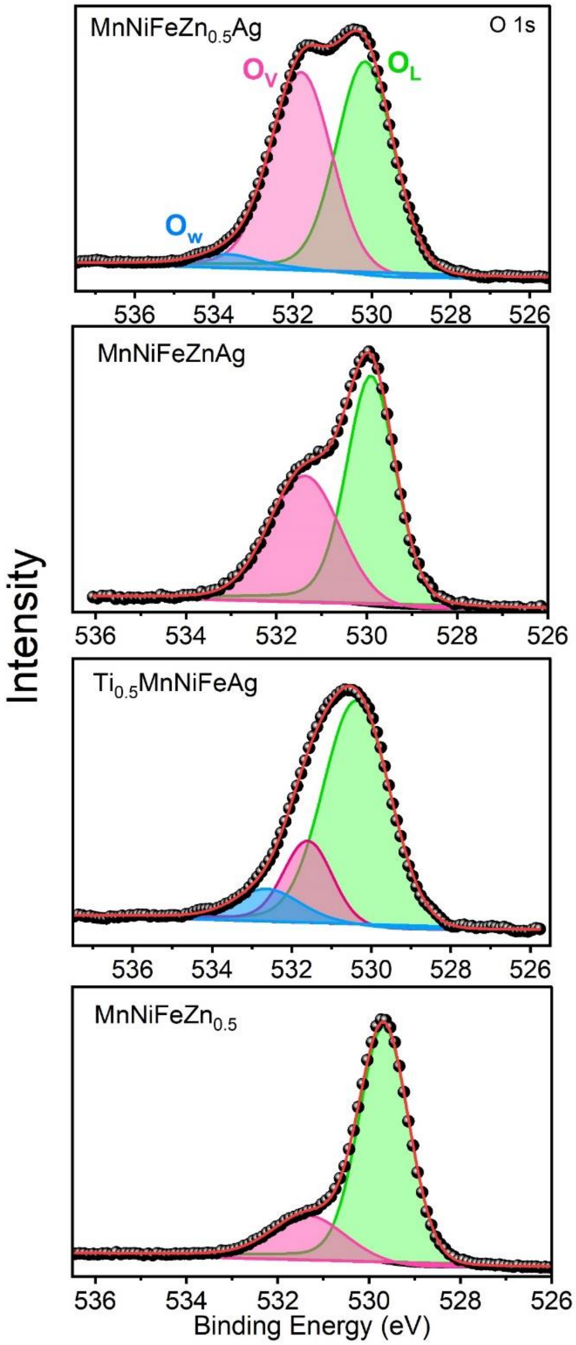

Figure S12. The O 1s XPS spectra for $MnNiFeZn_{0.5}Ag$ (AI22), MnNiFeZnAg (AI20), $Ti_{0.5}MnNiFeAg$ (AI23), and $MnNiFeZn_{0.5}$.

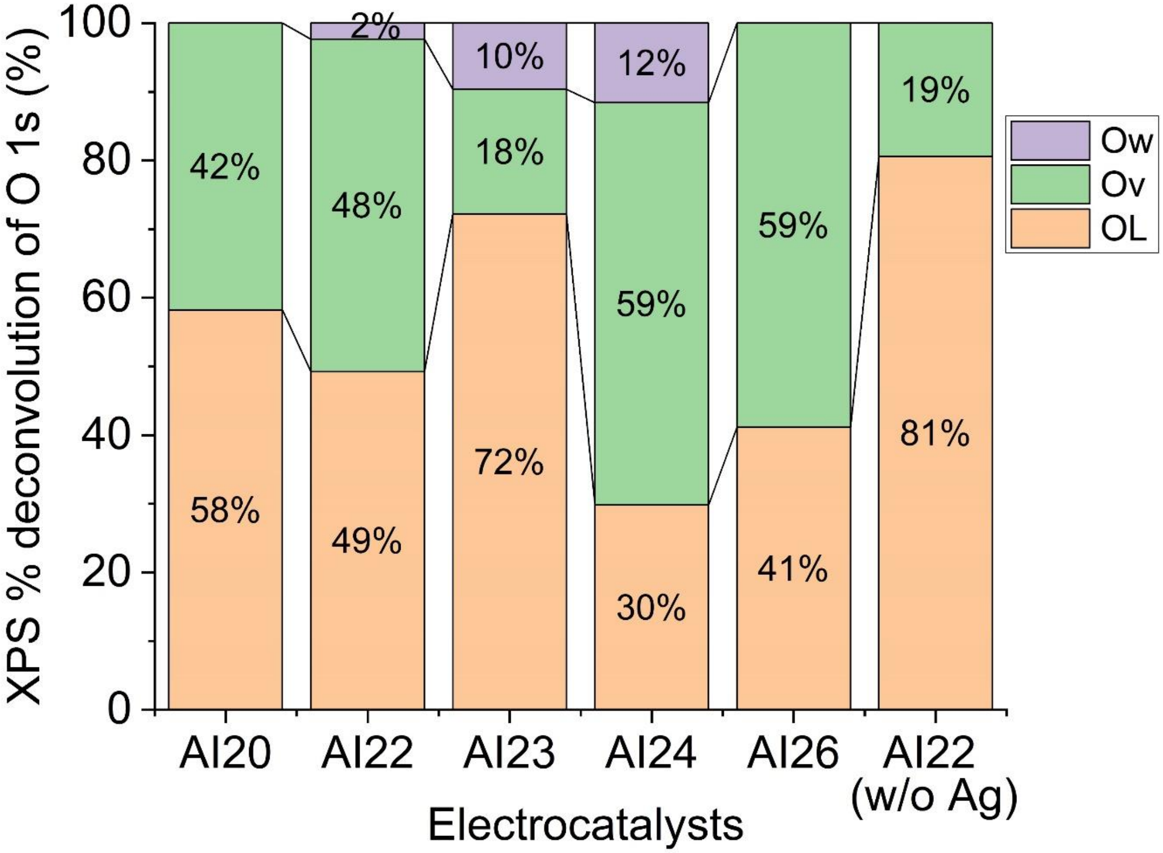

Figure S13. Peak area contribution of XPS deconvolution of O 1s in different electrocatalysts.

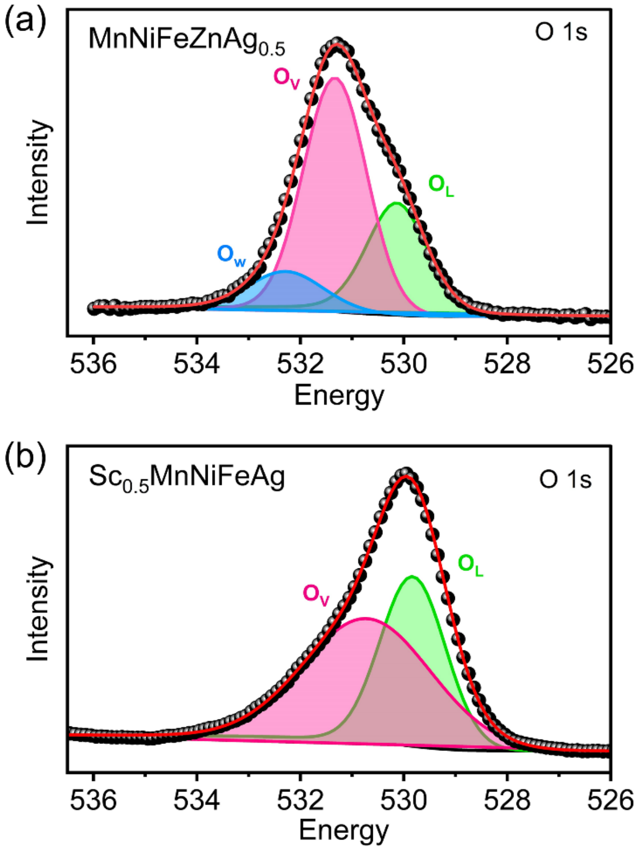

Figure S14. The O 1s XPS spectra for (a) $MnNiFeZnAg_{0.5}$ (AI24), and (b) $Sc_{0.5}MnNiFeAg$ (AI26).

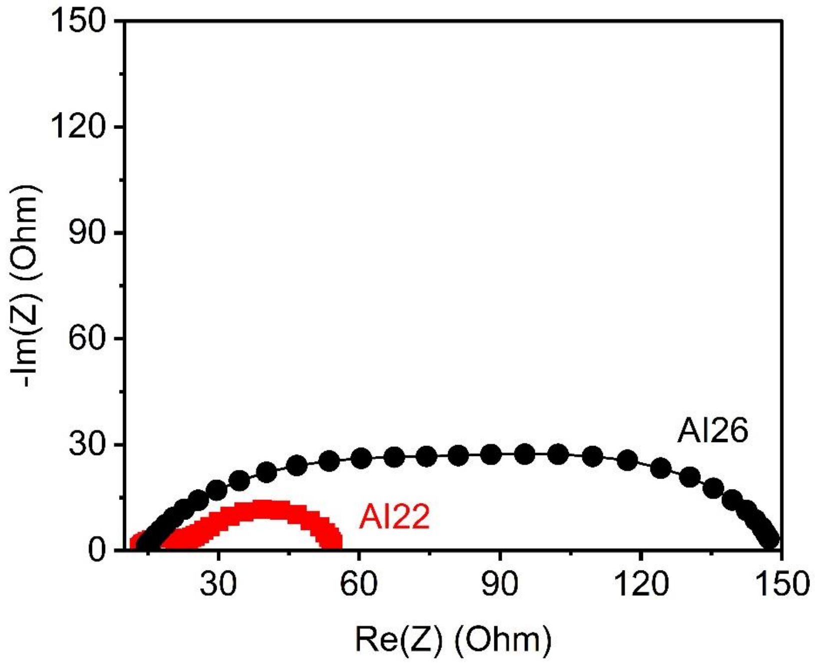

Figure S15. Nyquist plots obtained by EIS measurements. MnFeNiZn0.5Ag (AI22): R1 and $Sc_{0.5}$MnFeNiAg (AI26).

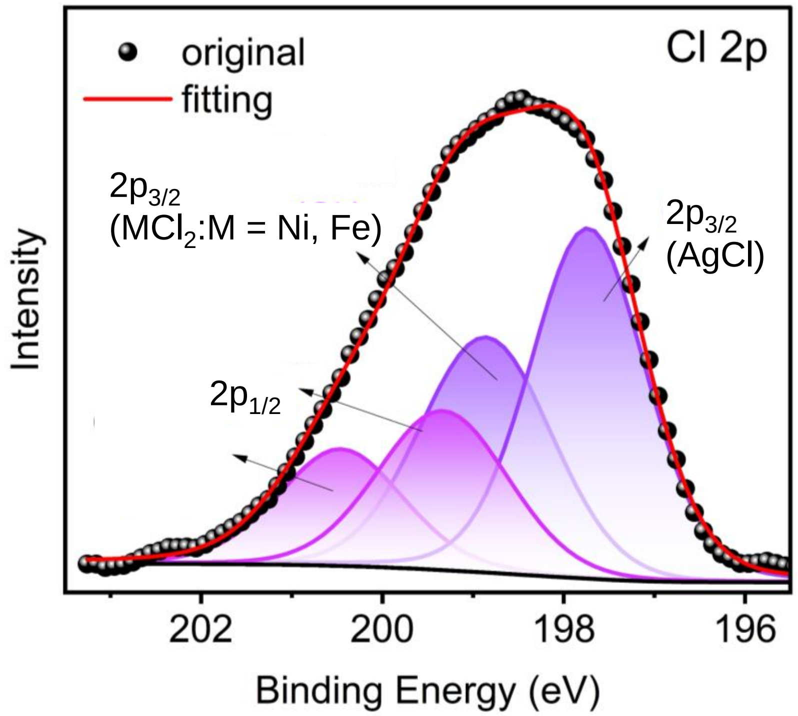

Figure S16. The Cl 2p XPS spectra for $MnNiFeZn_{0.5}Ag$ (AI22).

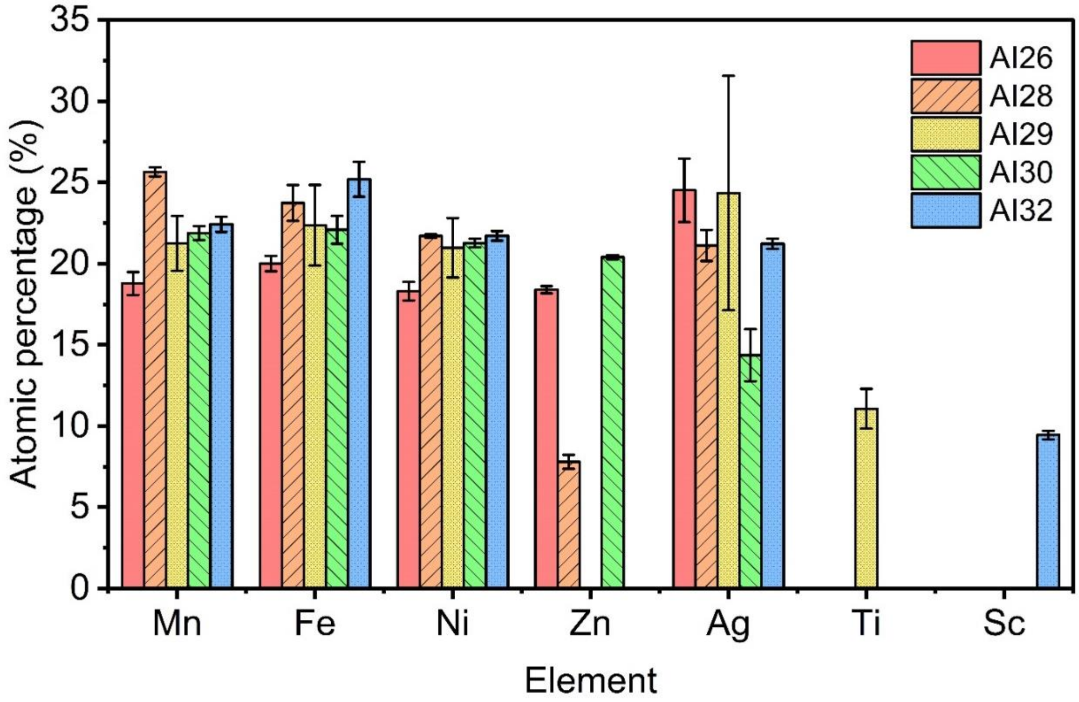

Figure S17. Elemental composition of the QEE samples: data collected from Table S2.

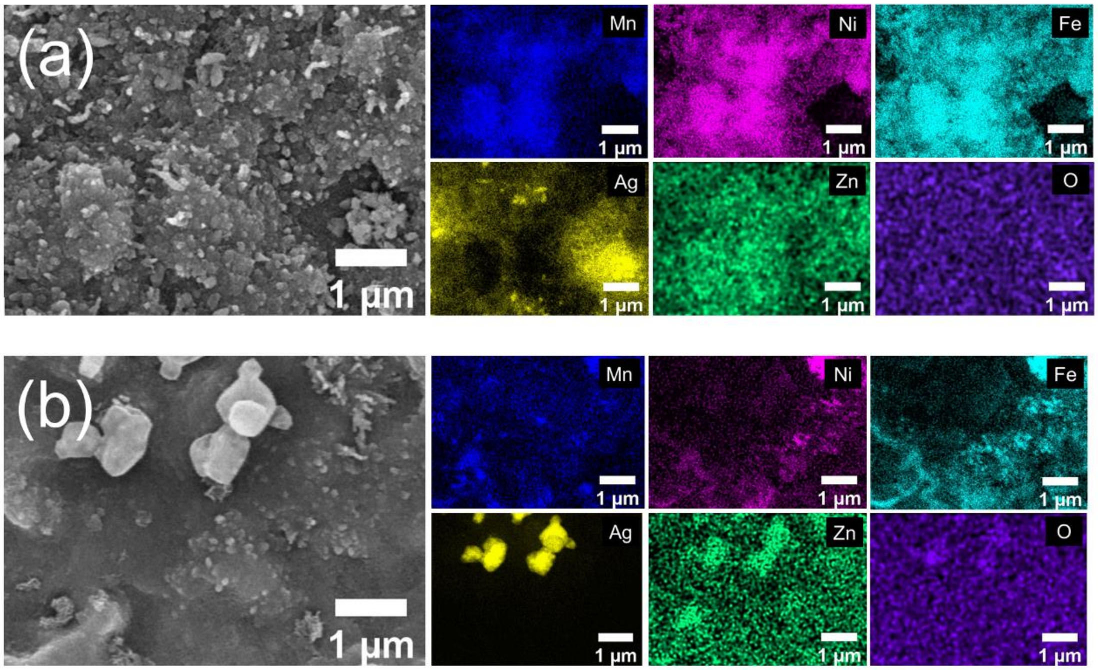

Figure S18. SEM images and the corresponding elemental EDS mapping of the $MnFeNiZn_{0.5}Ag$ (AI22) samples after electrochemical measurement in (a) 0.1 M KOH (pH 13), and (b) 0.1 M Na-Ci (pH 9.2).

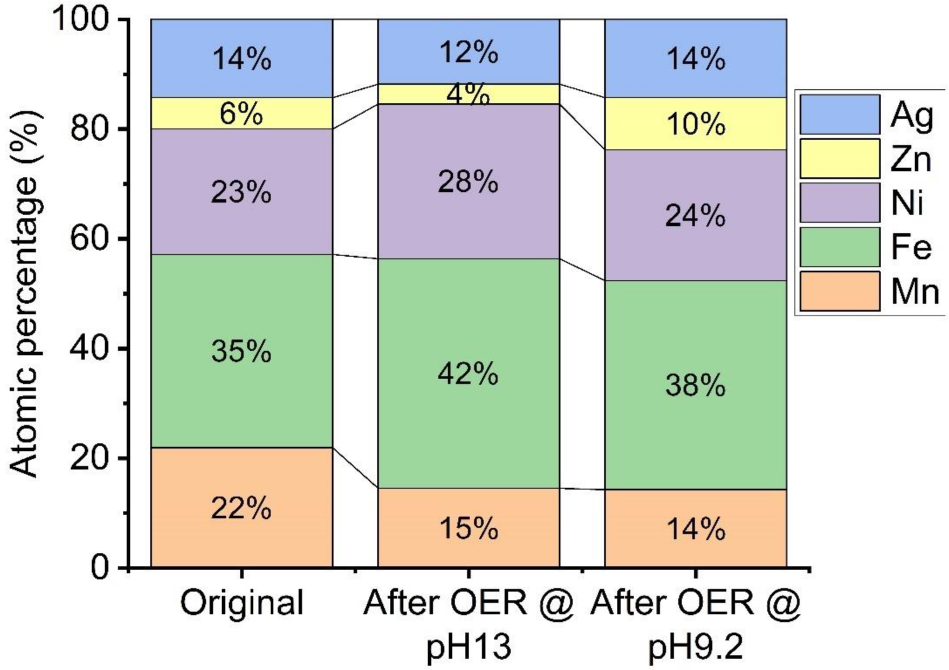

Figure S19. Elemental composition of the $MnFeNiZn_{0.5}Ag$ (AI22) samples estimated from the SEM-EDX mapping (Figure S5 and S16).

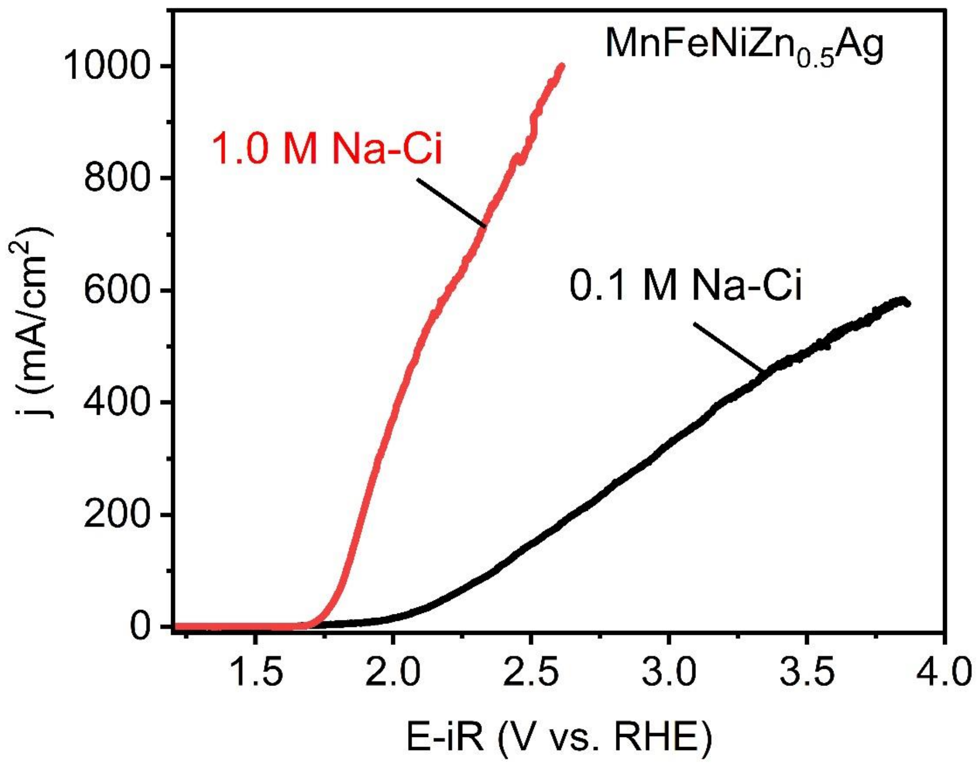

Figure S20 LSV curves of the $MnFeNiZn_{0.5}Ag$ (AI22) QEE samples in different electrolyte concentrations (pH 9.2); 0.1 M Na-Ci (black), and 1.0 M Na-Ci (red) at 80 ℃ with a scan rate of 1 mV/sec.

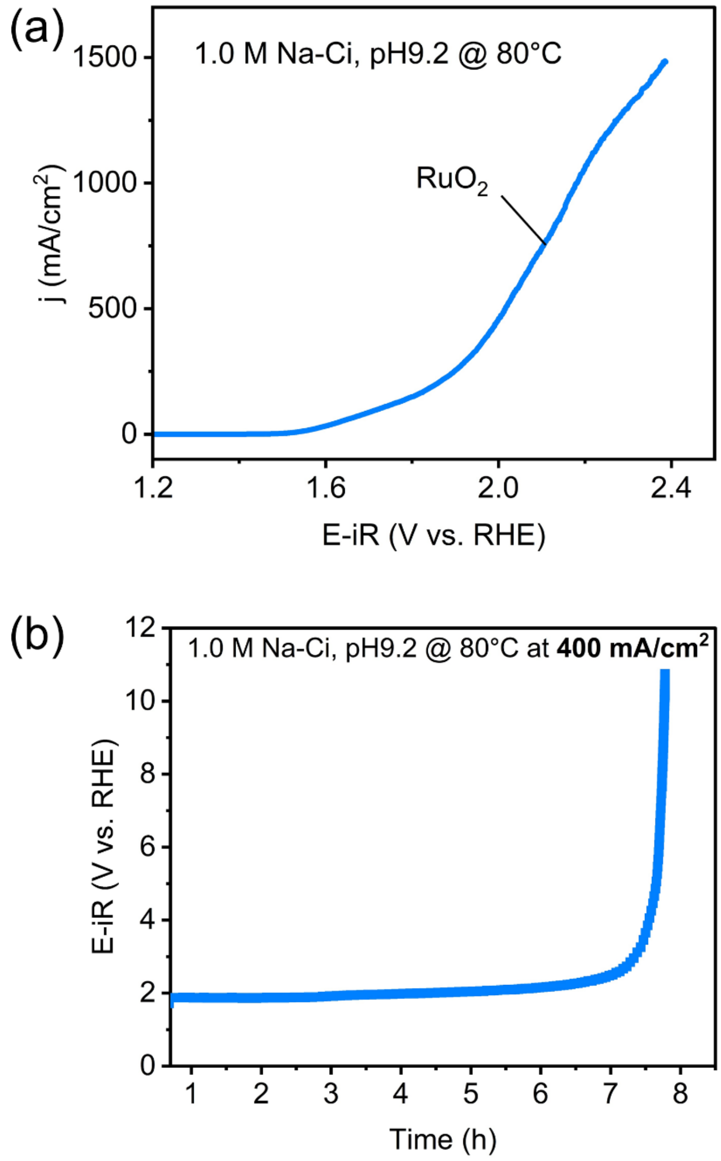

Figure S21. (a) LSV curve at a scan rate of 1 mV/sec and (b) CP curve of the RuO_2/Ti electrode at a high current density of 400 mA/cm^2 measured at 80 °C in 1.0 M Na-Ci electrolyte (pH 9.2).

Table S1. Elemental composition of the selected top-five QEE samples designed by se-AI.

Material ID	Activity Rank	Element1	Ratio	Element2	Ratio	Element3	Ratio	Element4	Ratio	Element5	Ratio
AI22	1	Mn	1	Fe	1	Ni	1	Zn	0.5	Ag	1
AI24	2	Mn	1	Fe	1	Ni	1	Zn	1	Ag	0.5
AI20	3	Mn	1	Fe	1	Ni	1	Zn	1	Ag	1
AI23	4	Ti	0.5	Mn	1	Fe	1	Ni	1	Ag	1
AI26	5	Sc	0.5	Mn	1	Fe	1	Ni	1	Ag	1

Table S2. Representative of random QEE candidate samples for AI training.

ID	Element 1	ratio	Element 2	ratio	Element 3	ratio	Element 4	ratio	Element 5	ratio
1	Sc	1	Fe	1	Ni	1	Zn	1	Zr	0.5
2	Ti	1	Mn	0.5	Fe	1	Ni	1	Ta	1
3	Mn	1	Fe	1	Ni	1	Cu	1	Zr	0.5
4	Sc	1	Mn	1	Zn	0.5	Zr	1	Ta	1
5	Ti	1	Fe	1	Ni	1	Zn	0.5	Zr	1
6	Ti	1	Fe	1	Cu	0.5	Zn	1	Ta	1
7	Fe	1	Ni	1	Zn	1	Sn	1	Ta	0.5
8	Sc	1	Mn	0.5	Ni	1	Zr	1	Sn	1
9	Fe	0.5	Cu	1	Zn	1	Ag	1	Sn	1
10	Sc	1	Mn	1	Zn	1	Zr	1	Ta	0.5

Table S3. Elemental composition of the QEE samples measured by ICP-OES analysis.

Element	Atomic percentage* (%)				
	AI 20	AI 22	AI 23	AI 24	AI 26
Mn	18.8	25.7	21.2	21.9	22.4
Fe	20.0	23.7	22.4	22.1	25.2
Ni	18.3	21.7	21.0	21.3	21.7
Zn	18.4	7.8	-	20.4	-
Ag	24.5	21.1	24.3	14.3	21.2
Ti	-	-	11.1	-	-
Sc	-	-	-	-	9.5

*Average atomic percentage for all samples (n = 3)

Table S4. Electrochemical properties comparison of the $MnFeNiZn_{0.5}Ag$ with other reported electrocatalysts.

Electrocatalysts	Electrolyte	pH	Current density (mA/cm^2)	η (mV)	Charge to mass ratio (C/g)	Temp. (°C)	Reference
$MnFeNiZn_{0.5}Ag$	1 M NaCi	9.2	10	493	2.43×10^9	80	This work
RuO_2	1 M NaCi	9.2	10	310	3.69×10^7	80	This work
Co-Ci-OH	1 M KCi	8.3	10	332	7.80×10^5	RT	S2
Ni_3N/Ni-Ci	1 M KCi	8.3	20	410	1.92×10^6	RT	S3
NiO/Ni-Ci	1 M KCi	8.3	15	387	1.00×10^6	RT	S4
Ni-Fe-Mg	0.5 M KCi	8.5	10	310	1.54×10^8	RT	S5
NiCo-Bi-LDH	0.1 M KBi	9.2	10	430	6.26×10^6	RT	S6
Co-Bi	0.1 M KBi	9.2	10	469	6.00×10^6	RT	S7
Co-Bi	0.1 M KBi	9.2	10	420	3.02×10^6	RT	S8
$CoFe_2O_4$/CoFeBi	0.1 M KBi	9.2	10	460	1.03×10^6	RT	S9
NiFe LDH	0.1 M KBi	9.2	10	376	1.36×10^6	RT	S10
NiFe LDH	0.1 M KBi	9.2	1	387	1.37×10^6	RT	S11
CuO	0.1 M KBi	9.2	1	-	7.20×10^4	RT	S12
CoP/CoBiPi	0.1 M KBi	9.2	10	410	1.62×10^6	RT	S13
CoFeBi/CoFe	0.1 M KBi	9.2	10	418	1.26×10^6	RT	S14
$Co(OH)_2$-Fe	1 M KBi+0.5 M KCl	9.2	500	-	1.36×10^7	80	S15
$CoFe(OH)_2$	1 M KBi+0.5 M KCl	9.2	500	-	1.54×10^7	80	S15
Ni_3Fe/Co, N-C	1.0 M NaCi	9.45	15	-	8.50×10^5	RT	S16
Fe-Ci	0.2 M KCi	9.75	10	560	1.62×10^7	RT	S17
$Fe_3O_4/NiFe_xO_y$	0.2 M KCi	9.75	1	410	9.00×10^3	RT	S18

Supporting Discussion 1: Material Characterization

To investigate the microscopic structure of QEEs, the scanning electron microscopy (SEM) was employed (**Figure S4a-e**)**.** These SEM images show that the materials consisting of Mn, Fe, Ni, Zn, and Ag (AI20, AI22, and AI24) exhibited a pronounced surface roughness with a presence of small nanoparticles, while the Ti-contained material of AI23 has a similar surface structure with a smaller nanoparticle than the other four materials. Surprisingly, a change in one element with Sc in the QEE material (AI26) significantly led to the drastic change in surface morphology: the nanopores and crack skeleton on the surface were formed in AI26 unlike to the other materials (**Figure S4e**). The crystal structures of QEEs were studied by X-ray diffractometry (**Figure S4f)**. The X-ray diffraction (XRD) patterns can be assigned to the crystal structures of a hexagonal-nickel oxide (space group: R-3m) with the cubic-silver chloride (space group: Fm-3m). In good agreement with the energy-dispersive X-ray (EDX) analysis applied along with SEM for the $MnFeNiZn_{0.5}Ag$ (**Figure S7**), all metal species along with oxygen were uniformly distributed in the surface indicating the formation of muti-component metal oxychloride material, but the silver and chloride were formed as the nanoparticles, shown as the cubic phase in the XRD pattern (**Fig. S4f**).

To investigate the further detailed on the QEE materials, a cross-section high resolution

transmission electron microscope (HR-TEM) was employed. The AI22 ($MnFeNiZn_{0.5}Ag$) material has an average thickness of 978.0 ± 139.8 nm (**Figure S7a**), which is thicker than other QEE samples, AI23 ($Ti_{0.5}MnFeNiAg$) for 288.7 ± 38.4 nm and AI26 ($Sc_{0.5}MnFeNiAg$) for 147.0 ± 24.4 nm (**Figure S8, S9)**. As for further details, the cross-section image shows that the AI22 material consists of small nanoparticles of AgCl as observed as black dots in the TEM image (**Figure S10**). Moreover, beside the AgCl crystals the AI22 material was found to be mainly amorphous or poorly crystalline by using selected area electron diffraction (SAED) (**Figure S5b,c**). The main SAED patterns can be assigned to the cubic structure of AgCl, which well-agreed with the XRD pattern (**Fig. S4f**). Annular dark-field scanning transmission electron microscopy (ADF-STEM) image and the elemental mapping to energy dispersive spectrometry (EDS) for the AI22 material ($MnFeNiZn_{0.5}Ag$) were shown in **Figure S5d-k**. The result of elemental mapping (**Fig. S5d-k**) again confirmed that this electrocatalyst have a homogeneous distribution of Mn, Fe, Ni, Zn, and O elements in the whole region. This homogeneous distribution is as same as for the other QEEs (AI23 and AI26, **Figs. S8 and S9**). Besides, the Ag element is mostly formed as nanoparticles and only small amount of Ag was found to be distributed to form the multicomponent metal oxide with other metal species.

Supporting Discussion 2: Electronic Properties of Transition Metals in QEE

The electronic property of the transition metals in the QEE materials is an important feature to reveal their activity through understanding the bonding between the metal active sites and the OER intermediates. High-resolution X-ray photoelectron spectrometry (XPS) was employed to determine the chemical composition and oxidation states of each element of the AI22 material. The coexistence of seven elements consisting of Mn, Fe, Ni, Zn, Ag, Cl and O in the AI22 QEE was confirmed in the survey XPS spectrum (**Figure S11**). The XPS spectrum of Mn 2p was presented in **Figure S6a**, where two oxidation states can be distinguished at 641.9 eV (Mn $2p_{3/2}$) and 653.6 eV (Mn $2p_{1/2}$) of Mn^{3+}, and at 646.7 eV (Mn $2p_{3/2}$) and 658.4 eV (Mn $2p_{1/2}$) of Mn^{4+}. Two different oxidation states were also found in the Fe 2p XPS spectrum (**Figure S6b**). The Fe^{2+} peaks were located at 711.0 eV (Fe $2p_{3/2}$) and 724.1 eV (Fe $2p_{1/2}$), while the Fe^{3+} peaks were located at 713.0 eV (Fe $2p_{3/2}$) and 726.1 eV (Fe $2p_{1/2}$) with the presence of their two clear satellite peaks. The Ni $2p_{3/2}$ spectrum shown in **Figure S6c** consisted of peaks at 856.2 eV and 858.9 eV corresponding to Ni^{2+} and Ni^{3+}, respectively. In addition, two clear satellite peaks at 862.3 eV (Ni^{2+}) and 865.0 eV (Ni^{3+}) can be observed. **Figure S6d** shows the Zn $2p_{3/2}$ spectrum, the peaks at 1022.0 eV and 1023.6 eV were contributed to Zn^{2+} corresponding to Zn-O and Zn-Cl, respectively. Besides, two oxidation states of Ag^{2+} (367.3 eV of Ag $3d_{5/2}$ and 373.3 eV of Ag $3d_{3/2}$) and Ag^{+}

(368.1 eV of Ag $3d_{5/2}$ and 374.1 eV of Ag $3d_{3/2}$) can be detected (**Figure S6e**). These results suggested that the five metal elements in the AI22 are presented with multiple valence states and mainly distributed with the high valence state species (e.g. Mn^{4+} or Ag^{2+}), which could lead to improve the OER activity of the AI22 material by forming a high active site density together with AgCl. **Figure S6f** shows the O 1s spectrum, which were deconvoluted into three components, the lattice oxygen of metal oxide (O_L) at 530.1 eV, the oxygen vacancy (O_v) at 531.8 eV, and the surface adsorbed oxygen/interlayer water (O_w) at 533.7 eV.[S19-21] The AI22 sample showed the high peak area contribution of O_v (48 at.% in the fraction of whole oxygen species) demonstrating a large amount of surface oxygen vacancies in comparison with the AI20, AI23, and $MnNiFeZn_{0.5}$ electrocatalysts, as shown in **Figure S12** and **S13**. In conjugation with the results of electrochemical properties (Figure 3), the results of XPS analysis suggest that the high density of O_v at the surface of AI22 which is a key for the high OER activity of this compound because O_v is known to work as an active site.[22-26] However, the AI26 QEEs shows the poor activity even exhibiting the highest O_v concentration of 59 at.% among the total oxygen species (**Figure S13** and **S14**) indicating that this electrocatalysts contain large numbers of active sites. The reason for the low electrochemical properties of AI26 will be due to the high charge transfer resistance as revealed by electrochemical impedance spectrometry (EIS) (**Figure S15**). It is known that the two

semicircles can be observed for DSA using Ti substrate. The first semicircle is corresponding to the charge transfer resistance of electrocatalyst itself and second corresponding to the charge transfer at an electrocatalyst/Ti substrate interface.[S27, 28] The EIS fitting result shows that AI26 has much larger resistances at both phases. This high resistance is probably due to the effect of Sc, which oxide phase shows an insulating nature. The XPS Cl 2p spectrum of the AI22 presented two oxidation states at 197.7 and 198.9 eV, mainly corresponding to Cl $2p_{3/2}$ of AgCl (**Figure S16**). Overall, a specific combination of five elements of Mn, Fe, Ni, Zn and Ag, was revealed to emerge a high OER activity by introducing a high active site density at the same time a high electronic conductivity. Especially, Zn or Sc seems to be a key to trigger a high Ov density, which could be induced by the coexistence of multiple elements with high valence states.

Supporting References

(S1) McCrory, C. C.; Jung, S.; Peters, J. C.; Jaramillo, T. F. Benchmarking heterogeneous electrocatalysts for the oxygen evolution reaction. *J. Am. Chem. Soc.* **2013**, *135*, 16977-16987.

(S2) Xie, M.; Yang, L.; Ji, Y.; Wang, Z.; Ren, X.; Liu, Z.; Asiri, A. M.; Xiong, X.; Sun, X. An amorphous Co-carbonate-hydroxide nanowire array for efficient and durable oxygen evolution reaction in carbonate electrolytes. *Nanoscale* **2017**, *9*, 16612-16615.

(S3) Xie, F.; Wu, H.; Mou, J.; Lin, D.; Xu, C.; Wu, C.; Sun, X. Ni_3N@Ni-Ci nanoarray as a highly active and durable non-noble-metal electrocatalyst for water oxidation at near-neutral pH. *J. Catal.* **2017**, *356*, 165-172.

(S4) Ma, M.; Liu, Y.; Ma, X.; Ge, R.; Qu, F.; Liu, Z.; Du, G.; Asiri, A. M.; Yao, Y.; Sun, X. Highly efficient and durable water oxidation in a near-neutral carbonate electrolyte electrocatalyzed by a core–shell structured NiO@Ni–Ci nanosheet array. *Sustain. Ener. Fuels* **2017**, *1*, 1287-1291.

(S5) Wang, N.; Cao, Z.; Zheng, X.; Zhang, B.; Kozlov, S. M.; Chen, P.; Zou, C.; Kong, X.; Wen, Y.; Liu, M.; et al. Hydration-Effect-Promoting Ni–Fe Oxyhydroxide Catalysts for Neutral Water Oxidation. *Adv. Mater.* **2020**, *32*, 1906806.

(S6) Chen, L.; Ren, X.; Teng, W.; Shi, P. Amorphous Nickel-Cobalt-Borate Nanosheet Arrays for Efficient and Durable Water Oxidation Electrocatalysis under Near-Neutral Conditions. *Chem. Euro. J.* **2017**, *23*, 9741-9745.

(S7) Yang, L.; Liu, D.; Hao, S.; Kong, R.; Asiri, A. M.; Zhang, C.; Sun, X. A cobalt-borate nanosheet array: an efficient and durable non-noble-metal electrocatalyst for water oxidation at near neutral pH. *J. Mater. Chem. A* **2017**, *5*, 7305-7308.

(S8) Ren, X.; Ge, R.; Zhang, Y.; Liu, D.; Wu, D.; Sun, X.; Du, B.; Wei, Q. Cobalt–borate nanowire array as a high-performance catalyst for oxygen evolution reaction in near-neutral media. *J. Mater. Chem. A* **2017**, *5*, 7291-7294

(S9) Ji, X.; Hao, S.; Qu, F.; Liu, J.; Du, G.; Asiri, A. M.; Chen, L.; Sun, X. Core–shell $CoFe_2O_4$@Co–Fe–Bi nanoarray: a surface-amorphization water oxidation catalyst operating at near-neutral pH. *Nanoscale* **2017**, *9*, 7714-7718.

(S10) Dong, Y.; Komarneni, S.; Wang, N.; Hu, W.; Huang, W. An in situ anion exchange induced high-performance oxygen evolution reaction catalyst for the pH-near-neutral potassium borate electrolyte. *J. Mater. Chem. A* **2019**, *7*, 6995-7005.

(S11) Dong, Y.; Komarneni, S.; Zhang, F.; Wang, N.; Terrones, M.; Hu, W.; Huang, W. "Structural instability" induced high-performance NiFe layered double hydroxides as oxygen evolution reaction catalysts for pH-near-neutral borate electrolyte: The role of intercalates. *Appl. Catal. B.* **2020**, *263*, 118343.

(S12) Liu, X.; Jia, H.; Sun, Z.; Chen, H.; Xu, P.; Du, P. Nanostructured copper oxide electrodeposited from copper(II) complexes as an active catalyst for electrocatalytic oxygen evolution reaction. *Electrochem. Commun.* **2014**, *46*, 1-4.
(S13) Cui, L.; Qu, F.; Liu, J.; Du, G.; Asiri, A. M.; Sun, X. Interconnected Network of Core–Shell CoP@CoBiPi for Efficient Water Oxidation Electrocatalysis under Near Neutral Conditions. *ChemSusChem* **2017**, *10*, 1370-1374.
(S14) You, C.; Ji, Y.; Liu, Z.; Xiong, X.; Sun, X. Ultrathin CoFe-Borate Layer Coated CoFe-Layered Double Hydroxide Nanosheets Array: A Non-Noble-Metal 3D Catalyst Electrode for Efficient and Durable Water Oxidation in Potassium Borate. *ACS Sustain. Chem. Eng.* **2018**, *6*, 1527-1531.
(S15) Komiya, H.; Shinagawa, T.; Takanabe, K. Electrolyte Engineering for Oxygen Evolution Reaction Over Non-Noble Metal Electrodes Achieving High Current Density in the Presence of Chloride Ion. *ChemSusChem* **2022**, *15*, e202201088.
(S16) Xu, B.; Wang, X.; Yang, X.; Chen, Z.; Sun, Y.; Liu, Q.; Li, C. Ni_3Fe nanoarray encapsulated in Co, N-dual doped carbon shell for efficient electrocatalytic oxygen evolution in both alkaline and carbonate electrolyte. *Electrochim. Acta* **2019**, *296*, 738-745.
(S17) Li, F.; Bai, L.; Li, H.; Wang, Y.; Yu, F.; Sun, L. An iron-based thin film as a highly efficient catalyst for electrochemical water oxidation in a carbonate electrolyte. *Chem. Commun.* **2016**, *52*, 5753-5756.
(S18) Luo, Z.; Martí-Sànchez, S.; Nafria, R.; Joshua, G.; de la Mata, M.; Guardia, P.; Flox, C.; Martínez-Boubeta, C.; Simeonidis, K.; Llorca, J.; et al. Fe_3O_4@$NiFe_xO_y$ Nanoparticles with Enhanced Electrocatalytic Properties for Oxygen Evolution in Carbonate Electrolyte. *ACS Appl. Mater. Int.* **2016**, *8*, 29461-29469.
(S19) Sathiya, M.; Rousse, G.; Ramesha, K.; Laisa, C. P.; Vezin, H.; Sougrati, M. T.; Doublet, M. L.; Foix, D.; Gonbeau, D.; Walker, W.; et al. Reversible anionic redox chemistry in high-capacity layered-oxide electrodes. *Nat. Mater.* **2013**, *12*, 827-835.
(S20) Li, Y.; Sun, Z.; Liu, D.; Gao, Y.; Wang, Y.; Bu, H.; Li, M.; Zhang, Y.; Gao, G.; Ding, S. A composite solid polymer electrolyte incorporating MnO_2 nanosheets with reinforced mechanical properties and electrochemical stability for lithium metal batteries. *J. Mater. Chem. A* **2020**, *8*, 2021-2032.
(S21) Dupin, J.-C.; Gonbeau, D.; Vinatier, P.; Levasseur, A. Systematic XPS studies of metal oxides, hydroxides and peroxides. *Phys. Chem. Chem. Phys.* **2000**, *2*, 1319-1324.
(S22) Mefford, J. T.; Rong, X.; Abakumov, A. M.; Hardin, W. G.; Dai, S.; Kolpak, A. M.; Johnston, K. P.; Stevenson, K. J. Water electrolysis on $La_{1-x}Sr_xCoO_{3-\delta}$ perovskite electrocatalysts. *Nat. Commun.* **2016**, *7*, 11053.

(S23) Fabbri, E.; Nachtegaal, M.; Binninger, T.; Cheng, X.; Kim, B.-J.; Durst, J.; Bozza, F.; Graule, T.; Schäublin, R.; Wiles, L.; et al. Dynamic surface self-reconstruction is the key of highly active perovskite nano-electrocatalysts for water splitting. *Nat. Mater.* **2017**, *16*, 925-931.
(S24) Grimaud, A.; Diaz-Morales, O.; Han, B.; Hong, W. T.; Lee, Y.-L.; Giordano, L.; Stoerzinger, K. A.; Koper, M. T. M.; Shao-Horn, Y. Activating lattice oxygen redox reactions in metal oxides to catalyse oxygen evolution. *Nat. Chem.* **2017**, *9*, 457-465.
(S25) Han, B.; Stoerzinger, Kelsey A.; Tileli, V.; Gamalski, Andrew D.; Stach, Eric A.; Shao-Horn, Y. Nanoscale structural oscillations in perovskite oxides induced by oxygen evolution. *Nat. Mater.* **2017**, *16*, 121-126.
(S26) Yoo, J. S.; Rong, X.; Liu, Y.; Kolpak, A. M. Role of Lattice Oxygen Participation in Understanding Trends in the Oxygen Evolution Reaction on Perovskites. *ACS Catal.* **2018**, *8*, 4628-4636.
(S27) Morita, M.; Iwakura, C.; Tamura, H. The anodic characteristics of manganese dioxide electrodes prepared by thermal decomposition of manganese nitrate. *Electrochim. Acta* **1977**, *22*, 325-328.
(S28) Morita, M.; Iwakura, C.; Tamura, H. The anodic characteristics of modified Mn oxide electrode: Ti/RuO_x/MnO_x. *Electrochim. Acta* **1978**, *23* 331-335.
